# Efficient Direct Nitrosylation of α-Diimine Rhenium Tricarbonyl Complexes to Structurally Nearly Identical Higher Charge Congeners Activable towards Photo-CO Release

**DOI:** 10.3390/molecules26175302

**Published:** 2021-08-31

**Authors:** Sara Nasiri Sovari, Isabelle Kolly, Kevin Schindler, Youri Cortat, Shing-Chi Liu, Aurelien Crochet, Aleksandar Pavic, Fabio Zobi

**Affiliations:** 1 Department of Chemistry, Fribourg University, Chemin Du Musée 9, 1700 Fribourg, Switzerland; sara.nasirisovari@unifr.ch (S.N.S.); isabelle.kolly@unifr.ch (I.K.); kevin.schindler@unifr.ch (K.S.); youri.cortat@unifr.ch (Y.C.); shing-chi.liu@unifr.ch (S.-C.L.); aurelien.crochet@unifr.ch (A.C.); 2 Institute of Molecular Genetics and Genetic Engineering, University of Belgrade, Vojvode Stepe 444a, 11042 Belgrade, Serbia; sasapavic@imgge.bg.ac.rs

**Keywords:** rhenium, dicarbonyl, nitrosyl, photoCORM

## Abstract

The reaction of rhenium α-diimine (N-N) tricarbonyl complexes with nitrosonium tetrafluoroborate yields the corresponding dicarbonyl-nitrosyl [Re(CO)_2_(NO)(N-N)X]^+^ species (where X = halide). The complexes, accessible in a single step in good yield, are structurally nearly identical higher charge congeners of the tricarbonyl molecules. Substitution chemistry aimed at the realization of equivalent dicationic species (intended for applications as potential antimicrobial agents), revealed that the reactivity of metal ion in [Re(CO)_2_(NO)(N-N)X]^+^ is that of a hard Re acid, probably due to the stronger π-acceptor properties of NO^+^ as compared to those of CO. The metal ion thus shows great affinity for π-basic ligands, which are consequently difficult to replace by, e.g., σ-donor or weak π-acids like pyridine. Attempts of direct nitrosylation of α-diimine *fac*-[Re(CO)_3_]^+^ complexes bearing π-basic OR-type ligands gave the [Re(CO)_2_(NO)(N-N)(BF_4_)][BF_4_] salt as the only product in good yield, featuring a stable Re-FBF_3_ bond. The solid state crystal structure of nearly all molecules presented could be elucidated. A fundamental consequence of the chemistry of [Re(CO)_2_(NO)(N-N)X]^+^ complexes, it that the same can be photo-activated towards CO release and represent an entirely new class of photoCORMs.

## 1. Introduction

The growing trend of antimicrobial resistance (AMR) poses a serious threat to the public’s health, making it more and more difficult to prevent and treat related diseases. Thousands of deaths have been attributed to AMR infections according to WHO findings published in 2018 [1], with an estimated death toll of millions of cases per year by 2050 if the appropriate measures are not taken [2]. With the increasing difficulty of major pharmaceutical companies to meet the demand of new antibiotics discovery and production, universities are contributing to the finding of new classes of active compounds. Pathogens, however, are likely to adapt rapidly and become resistant to new drugs if conventional medicinal chemistry approaches remain based exclusively on organic molecules, also in light of the fact that the majority of drugs in clinical development are modified versions of already-approved antibiotics [3].

There is an increasing awareness in academia of the potential of metal complexes to act as the new class of molecules for the purpose. Indeed, the unique chemistry and larger variety of 3D geometries of metal compounds can address targets and modes of action unavailable to organic molecules. In the last decade, complexes of virtually all transition metals have been evaluated [3,4], with rhenium, among others [5,6], showing promising potential for new antibiotic development [7,8,9,10].

Our group has been interested in the development of the chemistry of tri- and dicarbonyl rhenium complexes for applications in different medicinal fields [11,12,13,14,15,16,17,18], including their use as antibacterial agents. We recently reported studies on the antimicrobial properties of families of rhenium diimine (N-N) complexes and found several complexes of general formula *fac*-[Re^I^(CO)_3_(N-N)L]^+^ (L = pyridine, py, type-ligand), showing low to no toxicity in vivo and potent in vitro and in vivo activity against infection of clinically relevant bacteria (MRSA) and fungi (*Candida* species) [19,20]. Unlike similar complexes tested against cancers, where the overall charge of the compound does not seem to be a critical factor, a survey of the literature and our own data [20] indicates that positively charged rhenium complexes are most effective against the microbes. The mechanism of action of these agents remains largely unknown, but we hypothesize that the positive charge of the complexes is important for their interaction with phosphatidylglycerol and cardiolipin anionic lipids. All bacterial membranes contain at least 15% anionic lipid. Exposure to these lipids confers selectivity to cationic antimicrobial agents for toxicity against bacteria but not against mammalian cells [21,22]. Among the steps involved in the mechanism of action of the highly effective trimetallic complexes of Metzler-Nolte and Bandow, e.g., is the targeting of the cytoplasmic membrane, where the complexes affect membrane architecture and disrupt essential cellular functions, such as respiration and cell wall formation and integrity [7].

Given the data currently available on rhenium species, it is possible that by increasing the overall complex charge and by modulating molecules’ lipophylicity, binding to fungal and bacterial cell walls, cell membrane (and/or intracellular) accumulation might be enhanced, thereby potentially enhancing the overall antimicrobial efficacy of this class of molecules. To test this hypothesis, the new species should be structurally very close (if not identical) to the active *fac*-[Re^I^(CO)_3_(N-N)L]^+^ complexes, but with a higher charge. Charge modulation may be archived by the appropriate choice of a cationic N-N derivative or the monodentate ligand L in the drug sphere of the complexes [23], or by chemically modifying the *fac*-[Re^I^(CO)_3_]^+^ core. The latter possibility is more challenging, but there are options. In particular our attention turned to the equivalent *fac*-[Re^I^(CO)_2_(NO)]^2+^ core [24,25,26].

**Scheme 1:**
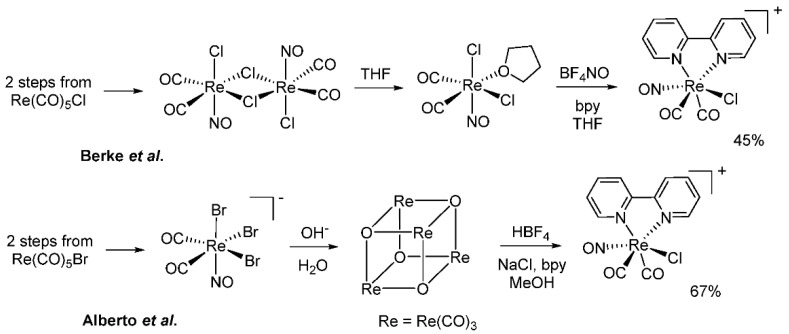
Known synthetic procedures to *fac*-[Re^I^(CO)_2_(NO)(N-N)(X)]^+^ type-complexes. The % yields refer to those of the last step only.

A compound of formula *fac*-[Re^I^(CO)_2_(NO)(bpy)Cl]^+^ (where *fac* refers here to the arrangement of the CO and NO ligands) was prepared previously by the groups of Alberto and Berke via a multi-step synthesis from cubic μ-oxo bridged teranuclear [Re(μ_3_-O)(CO)_2_(NO)]_4_ clusters [27] and, under inert conditions, from the [ReCl(μ-Cl)(CO)_2_(NO)]_2_ dimer respectively (Scheme 1) [28]. Nothing is known about the chemistry of the compound or if the same could be used as a synthon for *fac*-[Re^I^(CO)_2_(NO)(N-N)L]^2+^ species. We, therefore, set out to first establish a convenient synthetic procedure to *fac*-[Re^I^(CO)_2_(NO)(N-N)X]^+^ complexes (where X = halide) and then to study their substitution chemistry aiming at the realization of the dicationic species just mentioned above.

In this contribution, we describe our synthetic efforts, and we present an efficient direct nitrosylation of α-diimine rhenium tricarbonyl complexes widely applicable to different supporting N-N ligands. The resulting compounds are structurally nearly identical higher charge congeners of tricarbonyls. We found that, while similar complexes are reported as rhenium(I) dicarbonyl-nitrosyl species [24,25,26,27,28], the substitution chemistry of metal ion in *fac*-[Re(CO)_2_(NO)(N-N)X]^+^ is that of a hard Re acid, probably due to the stronger π-acceptor properties of NO^+^ as compared to those of CO. A fundamental consequence of this behavior is that *fac*-[Re(CO)_2_(NO)(N-N)X]^+^ complexes can be photo-activated towards CO release and represent an entirely new class of photoCORMs.

## 2. Results

### 2.1. Synthesis of fac-[Re(CO)_2_(NO)(N-N)X]^+^ Species

The chemistry of the *fac*-[Re(CO)_2_(NO)]^2+^ core is essentially dominated by cyclopentadienyl (Cp) species of general formula [CpRe(CO)_2_(NO)]^+^. These compounds are most often prepared by reaction of the corresponding [CpRe(CO)_3_] with either NO_2_PF_6_ [29], NOHSO_4_ [30], or NOBF_4_ [31,32]. The last two reagents were successfully used in the preparation of *fac*-[Re^I^(CO)_2_(NO)X_3_]^−^ [24,27], and of the two, the latter appeared to us the most convenient as the procedure involves simple addition of the nitrosonium tetrafluoroborate salt in a CH_2_Cl_2_ (DCM) solution of the rhenium tricarbonyl complex [32]. The reaction has also the advantage of producing an ion pair from the initially introduced components with, theoretically, no side products.

**Figure 1:**
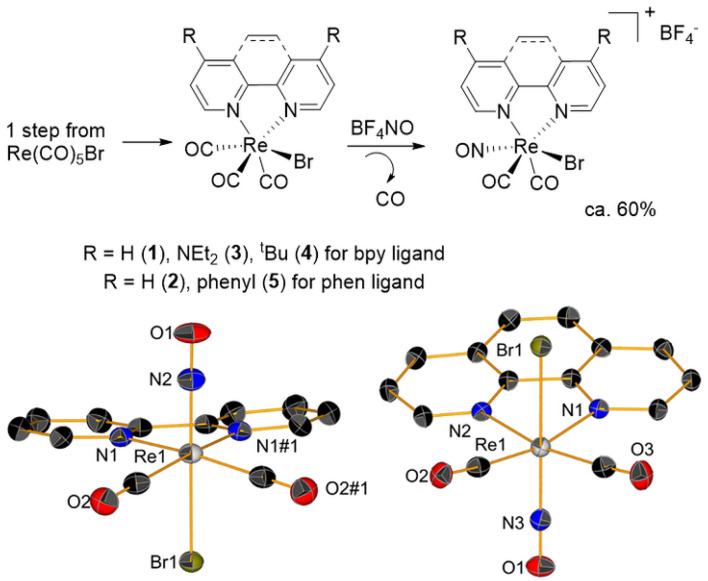
General synthetic approach to *fac*-[Re(CO)_2_(NO)(N-N)Br]^+^ complexes **1**–**5** (top) and crystal structures of compounds **1** (left) and **2**. Thermal ellipsoids are at 30% probability. Hydrogen atoms and BF_4_^−^ counter ion omitted for clarity. Selected bond lengths (Å): **1**, Re-N1 2.140(4), Re-N2 1.782(6), Re-Br 2.5372(7), Re-CO 1.990(5), C-O 1.123(6), N-O 1.164(7); **2**, Re-N1 2.154(3), Re-N2 2.153(3), Re-N3 1.801(4), Re-Br 2.5425(5), Re-CO 2.002(4) and 2.009(4), C-O 1.118(5) and 1.120(5), N-O 1.143(5). Selected bond angles (°): **1**, O1-N2-Re1 175.3(6); **2**, O1-N3-Re1 178.4(3).

Thus, we first attempted a reaction of *fac*-[Re^I^(CO)_3_(bpy)Br] with an excess (2.5-5 eq.) of NOBF_4_ in DCM. During the course of the reaction, the colour of the solution changed from a dark to a pale yellow and the same lost its photoluminescence properties. The corresponding dicarbonyl-nitrosyl *fac*-[Re(CO)_2_(NO)(bpy)Br]^+^ species (**1**) was isolated as a BF_4_^−^ salt in 70% yield (Figure 1), following crystallization by vapor diffusion with pentane or by slow evaporation of DMC, after excess NOBF_4_ was filtered off. In its crystalline form, **1** is slightly hygroscopic, and it decomposes if purification is attempted on a solid phase (silica or alumina). The same reaction was also attempted with phenanthroline (phen) and 4,4′- or 4,7- derivatives of bpy and phen, respectively, giving similar results (complexes **2**–**5**, Figure 1, in 60–70% yield). In comparison to the synthetic procedures of Alberto and Berke, our route appears straightforward, overall higher yielding and generally applicable to widely accessible α-diimine *fac*-[Re^I^(CO)_3_]^+^ species.

### 2.2. Spectroscopic Properties of fac-[Re(CO)_2_(NO)(N-N)X]^+^ Species

The physical properties of the *fac*-[Re(CO)_2_(NO)]^2+^ complexes prepared in this study are summarized in Table 1. The IR spectra of the compounds show the pattern expected for the dicarbonyl-nitrosyl species. Consistent with what reported before, the CO stretching (νCO’s) frequencies of *fac*-[Re(CO)_2_(NO)(N-N)Br]^+^ complexes **1**–**5** are substantially shifted to higher wavenumbers, which is unusual for carbonyls bound to the low valent metal [33,34]. In fact, the νCO’s are found in the region where rhenium(III) dicarbonyl complexes [35] (e.g., [Re^III^(CO)_2_Br_4_]^−^)[36] are observed, and much higher than corresponding rhenium(II) species [37]. The symmetric νCO mode of the molecules is actually not far from that of CO gas (2143 cm^−1^). The evidence points to a significant reduction of Re-CO π-backbonding in favour of the stronger π-acceptor NO^+^. The stretching vibration of the NO^+^ ion usually occurs in the 2300–2350 cm^−1^ frequency range, e.g., 2340 cm^−1^ in NOBF_4_ [38], 2326 cm^−1^ in NOAuF_6_ [39], and 2298 cm^−1^ in sulfuric acid solutions [40]. Its vibrational frequency in the complexes is found in the 1800 cm^−1^ region, closer to that of a N=O double bond than that of the initial triple bond. This indicates that the π*p(NO)-orbitals accept electron density to a great degree, depleting the metal ion of the same. Theoretically, a N=O double implies full occupation of a π*p(NO)-orbital, which would formally result in a metal center of higher oxidation state. Indeed, this formalism could account for the reactivity of the species (*vide infra*) and their lack of photoluminescence (see Supplementary Materials).

**Figure 2:**
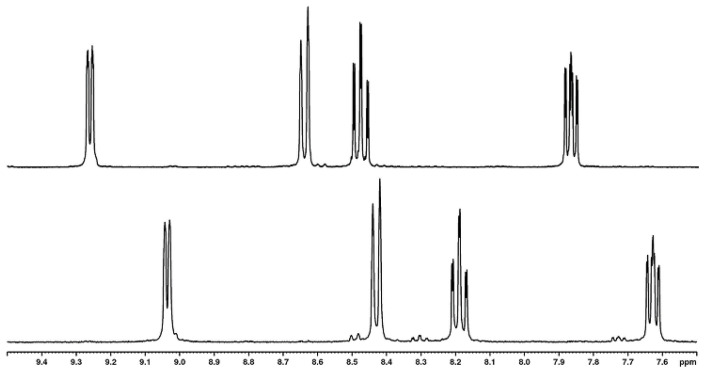
Aromatic regions of the ^1^H-NMR spectra of *fac*-[Re(CO)_2_(NO)(bpy)Br]^+^ (**1**, top) and *fac*-[Re^I^(CO)_3_(bpy)Br]^+^. Both spectra recorded in CD_3_CN.

The UV-Vis spectra of the compounds are characterized by a main absorption with two closely spaced maxima in 300–340 nm region (Table 1 and Supplementary Materials). Only complexes **2** and **5** (phenanthroline derivatives) show a single relatively broad peak for the same absorption, with two additional lower-lying small peaks at 340 and 375 nm, respectively. We did not perform a TDDFT analysis, but we suggest that the high-energy transitions are likely associated with π → π* intra-ligand transitions attributed to the diimine-system. In solution, all dicarbonyl-nitrosyl species show sharp signals in their NMR spectra, consistent with the diamagnetic nature of the molecules. With respect to the corresponding tricarbonyl complexes, *fac*-[Re(CO)_2_(NO)(N-N)X]^+^ show consistently a downfield shift of the N-N signals (Figure 2 and Supplementary Materials). This evidence further supports the assignment of a higher oxidation state of the Re atom.

### 2.3. Attempted Synthesis of fac-[Re(CO)_2_(NO)(N-N)L]^2+^ Species

**Figure 3:**
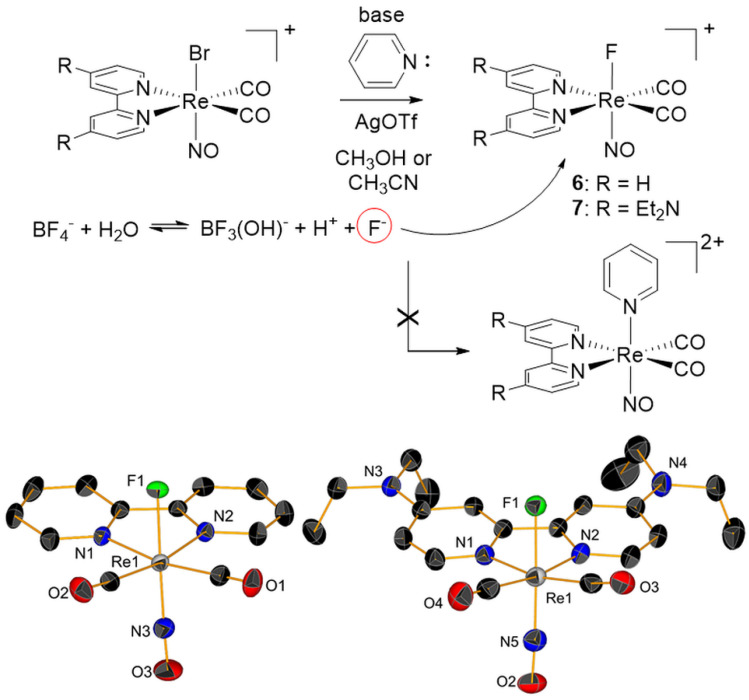
Reaction scheme of attempted synthesis *fac*-[Re(CO)_2_(NO)(N-N)py]^2+^ species from corresponding dicarbonyl-nitrosyl species in the presence of Ag^+^ and py (top) and crystal structure of compound **6** (left) and **7**. Thermal ellipsoids are at 30% probability. Hydrogen atoms and BF_4_^−^ counter ion omitted for clarity. Selected bond lengths (Å): **6**, Re-N1 2.138(4), Re-N2 2.145(4), Re-N3 1.773(6), Re-F 1.970(3), Re-CO 2.020(6) and 1.999(6), C-O 1.108(7) and 1.115(7), N-O 1.159(7); **7**, Re-N1 2.121(3), Re-N2 2.123(4), Re-N5 1.763(4), Re-F 1.984(3), Re-CO 2.017(5) and 1.991(6), C-O 1.123(6) and 1.129(6), N-O 1.185(5). Selected bond angles (°): **6**, O3-N3-Re1 177.3(6); **7**, O2-N5-Re1 176.8(4).

Having established a sufficiently high-yielding procedure for *fac*-[Re(CO)_2_(NO)(N-N)Br]^+^ ions, we moved to explore the substitution of Br^−^ for neutral pyridine (py) ligands. Typically, for the corresponding tricarbonyl complexes, bromide replacement is achieved either by treatment with trifluoromethanesulfonic acid or by addition of a silver salt. We first monitored by ^1^H-NMR the addition of AgCF_3_SO_3_ to *fac*-[Re(CO)_2_(NO)(bpy)Br]^+^ (**1**) in coordinating wet solvents like CH_3_OH or CH_3_CN. The spectrum revealed no change in the frequency of the proton signals over a period of 3 days. In a parallel reaction, the same conditions were used, but pyridine was added to the mixture. Overnight, new signals appeared in the NMR spectrum, but the set of frequencies attributable to free py remained unchanged. We initially hypothesized that the hydroxide *fac*-[Re(CO)_2_(NO)(bpy)(OH)]^+^ had formed, but later found that py, as a base, promoted BF_4_^−^ hydrolysis to [BF_3_(OH)]^−^ and F^−^ [41], and the latter substituted Br^−^ to give *fac*-[Re(CO)_2_(NO)(bpy)F]^+^ (**6**, Figure 3). As mentioned above, this type of reactivity of the metal ion is consistent with a hard Re acid and fully congruous with the spectroscopic data discussed above.

The higher apparent formal oxidation state of the metal ion implies the depletion of electron density from the same, and affinity for π-basic ligands rater σ-donors. We reasoned, therefore, that electron-donating substituents on N-N might help stabilize the rhenium ion towards the same reaction. NR_2_ groups (where R = aliphatic chain) are amongst the most effective electron donating groups [42]. However, when the *N*^4^,*N*^4^,*N*^4′^,*N*^4′^-tetraethyl-[2,2′-bipyridine]-4,4′-diamine (Et_2_N-bpy) complex **3** was used the corresponding *fac*-[Re^I^(CO)_2_(NO)(Et_2_N-bpy)F]^+^ (**7**) also formed as the only product. Compound (**7**) was isolated in 30% yield and was recrystallized from methanol. Its X-ray structure is also shown in Figure 3.

**Figure 4:**
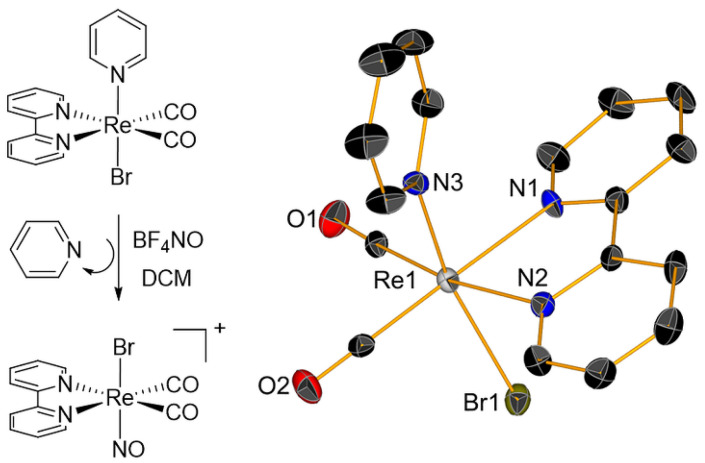
Reaction scheme of attempted synthesis *fac*-[Re(CO)_2_(NO)(N-N)py]^2+^ species from corresponding [Re^I^(CO)_2_(N-N)(py)Br] complex **8** and crystal structure of the same. Thermal ellipsoids are at 30% probability. Hydrogen atoms are omitted for clarity.

We recently published synthetic procedures to aerobically stable and substitutionally labile α-diimine rhenium(I) dicarbonyl complexes of formula [Re^I^(CO)_2_(N-N)Br(py)] capable of exchanging the halide for other ligands [37]. Therefore, we next tried the reaction of the corresponding [Re^I^(CO)_2_(bpy)Br(py)] complex (**8**) with NOBF_4_. Under reaction conditions similar to those applied for the synthesis of **1**–**5**, complex **8** reacted by substituting py for NO^+^ giving **1** (Figure 4). We only found trace evidence in the MS spectrum of the desired *fac*-[Re(CO)_2_(NO)(bpy)py]^2+^ species.

It should be noted here that Rattat reported that whereas the imidazole (Im) complexes [ReCl_2_(Im)(CO)_2_(NO)] and [ReCl(Im)_2_(CO)_2_(NO)]^+^ can be synthesized in high yields from [NEt_4_][ReCl_3_(CO)_2_(NO)] or [ReCl(μ-Cl)(CO)_2_(NO)]_2_, isolation of the [Re(Im)_3_(CO)_2_(NO)]^2^^+^ was not successful [43]. The authors argued that this is due to the behaviour of the *fac*-[Re(CO)_2_(NO)]^2+^ fragment in water (the reaction solvent), where the core initially binds three water molecules, one of which is deprotonated, and the resulting hydroxy group cannot be substituted by the σ-donor Im [44]. However, the trace evidence of the desired *fac*-[Re(CO)_2_(NO)(bpy)py]^2+^ species we detected by mass spectrometry, encouraged us to pursue our attempts.

**Figure 5:**
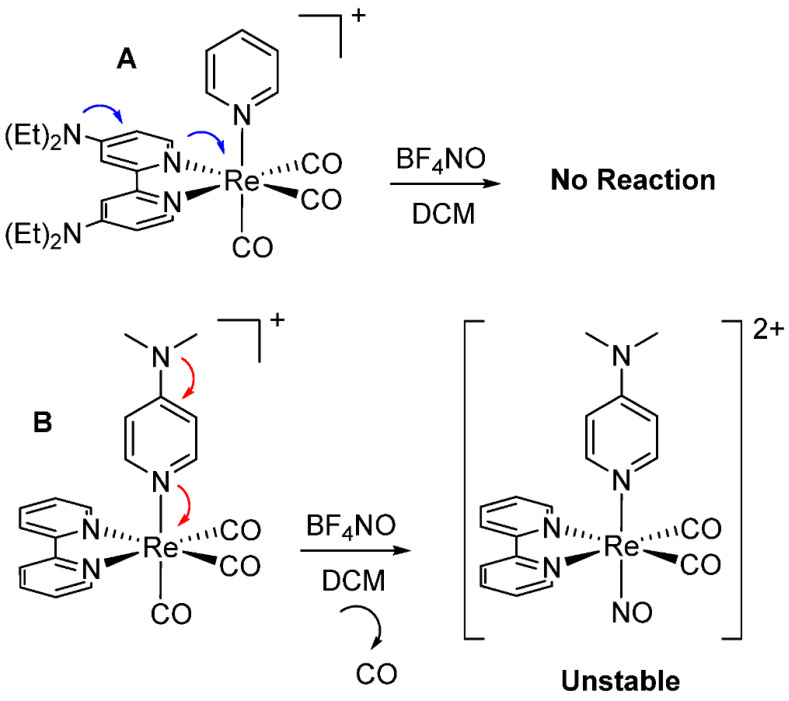
Molecular possibilities (**A**,**B**) envisioned to stabilize the *fac*-[Re(CO)_2_(NO)]^2+^ core via EDS on N-N and monodentate py ligand and reactions of corresponding tricabonyl complexes with NO^+^.

Therefore, we probed directly the reactivity of *fac*-[Re^I^(CO)_3_(N-N)(py)]^+^ complexes with nitrosonium tetrafluoroborate. Following the logic above, we probed the reaction of *fac*-[Re(CO)_3_]^+^complexes with combinations of bpy and Et_2_N-bpy (as N-N ligands) with py and *N*,*N*-dimethylpyridin-4-amine (Me_2_N-py) [45,46]. What guided our choice, was again consideration that electron-donating substituents (EDS) on N-N or py might be needed to stabilize the rhenium ion in the particular ligand arrangement. We envisioned two cases as shown in Figure 5. In case **A** we considered the possibility of EDS on the bidentate N-N ligand *cis* to NO, in case **B** EDS on the monodentate ligand trans to NO. As illustrated in Figure 5, we found no evidence of reaction with NO^+^ when the *fac*-[Re^I^(CO)_3_(Et_2_N-bpy)(py)]^+^ complex **A** was tested. However, complex **B** gave the corresponding *fac*-[Re(CO)_2_(NO)(bpy)(Me_2_N-py)]^2+^ (**9**) species as the kinetic, but not as the thermodynamically stable, product. The dicationic species could be isolated as it precipitated immediately from DCM following CO replacement by NO^+^. Evidence for the product came from both NMR and IR, which show, respectively, the expected downfield shift of all protons and the dicarbonyl-nitrosyl pattern (Supplementary Materials). The complex, however, is not pure and purification or crystallization attempts invariably lead to its decomposition.

### 2.4. Reactivity of fac-[Re^I^(CO)_3_(N-N)OR] Species (OR = π-Base) with NO^+^

While studying the reactions above, and in order to better understand the chemistry of the NO^+^ species, we also decided to explore nitrosylation of *fac*-[Re^I^(CO)_3_(N-N)] complexes with monodentate π-basic ligands (OR). Given the apparent affinity of the *fac*-[Re(CO)_2_(NO)]^2+^ core for π-donors, we reasoned that *fac*-[Re^I^(CO)_3_(N-N)OR] species might offer us a different chemical approach for the design of an efficient synthetic strategy to *fac*-[Re(CO)_2_(NO)(N-N)L]^2+^ compounds. Therefore, we decided to test the reaction of *fac*-[Re^I^(CO)_3_(N-N)OR], where OR = OH^−^, benzoate (**10**), 2-(4-bromophenyl)acetate (**11**).

**Figure 6:**
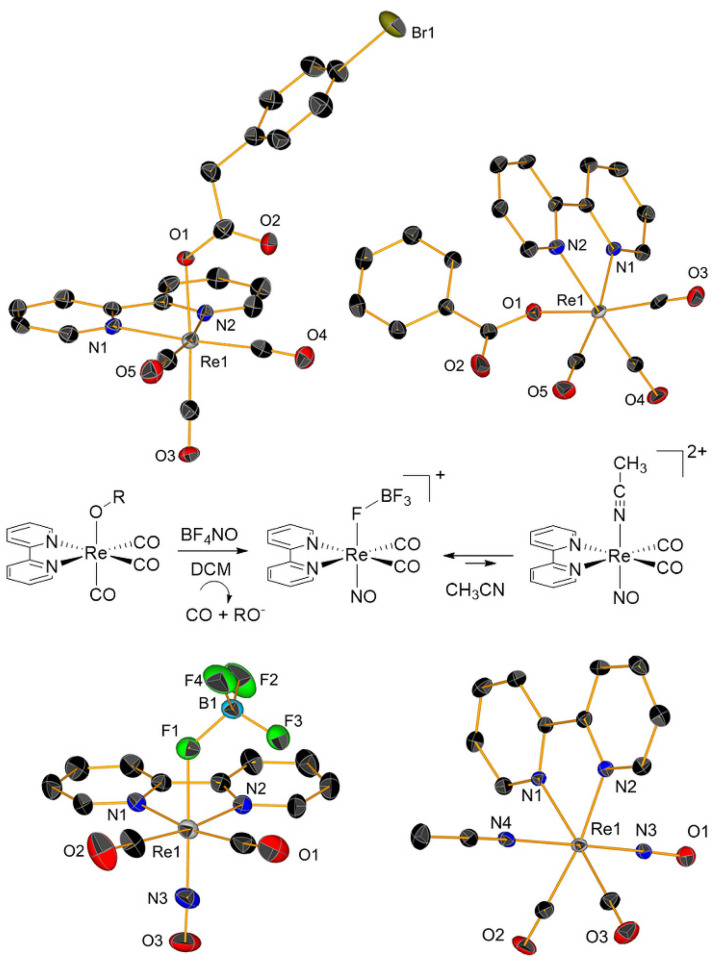
Top: crystal structures of carboxylato compounds **10** (left) and **11**. Middle: scheme of the reaction of *fac*-[Re^I^(CO)_3_(N-N)(OR)] (OR = OH^−^, benzoate, 2-(4-bromophenyl)acetate) with NOBF_4_. Bottom: Bottom: crystal structures of **12** (left) and the *fac*-[Re(CO)_2_(NO)(N-N)(CH_3_CN)]^2^^+^ ion (**13**). Thermal ellipsoids are at 30% probability. Selected bond lengths (Å) for **12**: Re-F1 2.082(4), Re-N1 2.137(6), Re-N2 2.132(5), Re-N3 1.794(7), Re-CO 1.999(9) and 1.985(9), C-O 1.123(10) and 1.125(10), N-O 1.139(8); for **13**: Re-N1 2.145(5), Re-N2 2.139(6), Re-N3 1.791(5), Re-N4 2.079(6), Re-CO 1.991(7) and 1.997(8), C-O 1.118(9) and 1.123(9), N-O 1.151(7). Selected bond angles (°): **12**, O3-N3-Re1 177.8(8); **13**, O1-N3-Re1 177.5(5).

To our surprise, irrespective of the OR ligand, nitrosylation of *fac*-[Re^I^(CO)_3_(N-N)OR] gave always the same dicarbonyl-nitrosyl product (i.e., with the identical spectroscopic signature). In the case of OR = benzoate or 2-(4-bromophenyl)acetate, the NMR evidence clearly indicated that OR was no longer coordinated to the rhenium ion. However, it was initially difficult to reconcile the spectroscopic fingerprints of the product with those of the same obtained from the hydroxo complex (i.e., when OR = OH^−^), also in light of the fact that we used dry solvents in the manipulations. The relatively high νCO frequencies of the product (2127 and 2069 cm^−1^) were also surprising and, on the basis of what we have shown before [33,34], they could not correspond to the electronic contribution of a coordinated OH^−^ base. Kurz and Alberto showed that the reactivity μ-oxo bridged teranuclear [Re(μ_3_-O)(CO)_2_(NO)]_4_ clusters towards neutral bidentate ligands is possible by disassembly of the same in HBF_4_ and proceeds “presumably [*via*] Re-bound labile BF_4_^−^ anion” [27]. We managed to crystallize the nitrosylated product obtained from *fac*-[Re^I^(CO)_3_(N-N)OR] and we confirmed that the reaction gives *fac*-[Re(CO)_2_(NO)(N-N)(BF_4_)][BF_4_] (**12**) as the only product in good yield (Figure 6). To our knowledge, **12** is only the fourth structurally characterized Re-FBF_3_ complex, and the very first one of its kind [47,48,49]. The Re-FBF_3_ bond is persistent in solution, and in CH_3_CN only a small fraction of the molecules (ca. 5–7%) substitute the anion for CH_3_CN. We were also able to selectively crystallize out the *fac*-[Re(CO)_2_(NO)(N-N)(CH_3_CN)]^2^^+^ complex (**13**) as a [Na(BF_4_)_3_]^2-^ salt (Figure 6), but we did not study it further.

### 2.5. X-ray Crystallography

**Figure 7:**
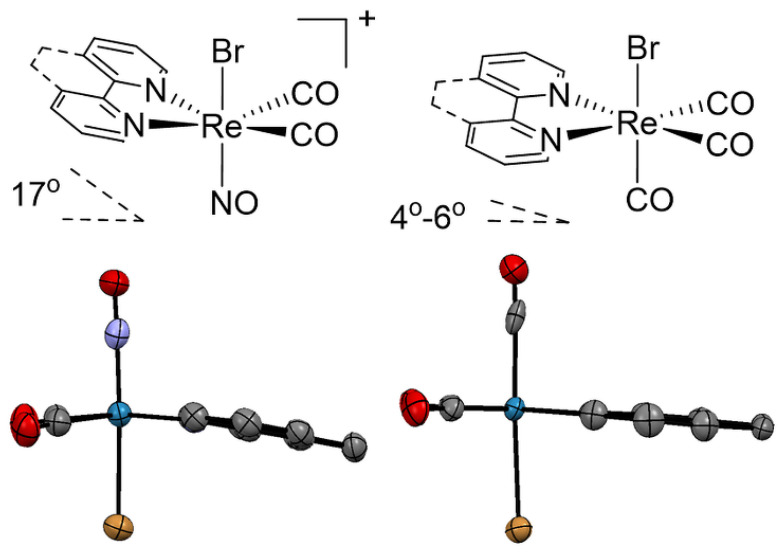
Main structural differences between related diimine *fac*-[Re(CO)_2_(NO)]^2+^ and *fac*-[Re(CO)_3_]^+^ species. At the bottom we compare the structures of **2** and a tricarbonyl phen derivative [53].

Crystallographic details of all complexes reported here are in Supplementary Materials, while selected bond lengths of *fac*-[Re(CO)_2_(NO)]^2+^ species are given in figure captions. All rhenium dicarbonyl-nitrosyl complexes show a distorted octahedral geometry around the Re ion. Structural analysis of the species and comparison to related *fac*-[Re(CO)_3_]^+^ species, revealed the following general characteristics. Within 3σ, the Re-CO and the C≡O bonds are respectively longer and shorter than the corresponding tricarbonyl complexes. There is no significant statistical difference in the Re-Br bond, while the Re-N(diamine) is slightly shorter in *fac*-[Re(CO)_2_(NO)]^2+^ species. In these, overall, crystal parameters are consistent with a rhenium ion in a higher oxidation state than +1 and are in agreement with the spectroscopic data. The shorter C≡O distances, e.g., are clearly reflected in the higher frequency of vibration of the bond in the IR spectrum. Likewise, the longer N-O distance (by ca. 0.1 Å in comparison to the free ion [50,51]) of Re-bound NO is observed in a lower frequency vibration of the same. Perhaps the most striking feature in *fac*-[Re(CO)_2_(NO)(N-N)Br]^+^ species (**1**–**5**) is represented by the bending (ca. 17°, but up to 25° for complex **7**) of the diamine ligand away from the bound NO (the diimine and the Re(CO)_2_ planes define the angle). In comparison, the same angle measures 4–6° in corresponding tricarbonyl molecules, and ca. 11° for **12** and **13**. Similarly, in nitrosyl complexes **1**–**5**, the two COs are also slightly bent towards the same direction (Figure 7). Overall, the molecules appear to be moving towards a trigonal distortion observed in octahedral d^4^ complexes [52].

### 2.6. CO Releasing Properties

**Figure 8:**
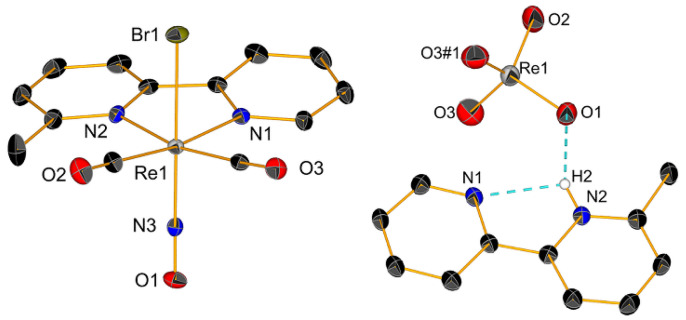
Crystal structures of compounds **14** (left) and its fully oxidized product. Thermal ellipsoids are at 30% probability. Hydrogen atoms and BF_4_^−^ counter ion omitted for clarity. Selected bond lengths for **14** (Å): Re-N1 2.135(4), Re-N2 2.188(4), Re-N3 1.784(4), Re-Br 2.5358(5), Re-CO 2.006(5) and 1.975(5), C-O 1.120(6) and 1.135(6), N-O 1.161(5). Selected bond angles (°): **14**, O1-N3-Re1 177.8(4).

In the initial phases of our investigation, we also probed the direct nitrosylation of tricarbonyl complexes with *ortho*-substituted α-diimines. The reaction is generally lower yielding with these ligands, but it works as well. In synthetic terms, nitrosylation of the complex bearing 6-methyl-2,2′-bipyridine (6Me-bpy) gave the best results (67% yield). We found, however, that when CH_3_CN solutions of *fac*-[Re(CO)_2_(NO)(6Me-bpy)Br]BF_4_ (**14**) were left exposed to ambient light, over time, crystals of the fully oxidized perrhenate ion appeared (Figure 8).

This observation indicated to us either that steric hindrance at the *ortho* position of the α-diimine ligand can destabilize *fac*-[Re(CO)_2_(NO)]^2+^ species which can then be oxidized by O_2_ to ReO_4_^−^, or that the complexes are photo-labile towards CO (and/or NO) release, i.e., acting as photoCORMs. Marti has shown that, unlike the tricarbonyl congener, the *fac*-[Re(CO)_2_(NO)Br_3_]^−^ ion reacts with tetradentate chelating ligands giving monocarbonyl-mononitrosyl species, indicative of a tendency of the rhenium dicarbonyl-nitrosyl complexes to liberate CO if specific conditions are satisfied [25]. Consequently we probed the CO-releasing properties of the *fac*-[Re(CO)_2_(NO)(N-N)X]^+^ molecules under conditions of the standard myoglobin (Mb) assay in the presence and absence of light.

**Figure 9:**
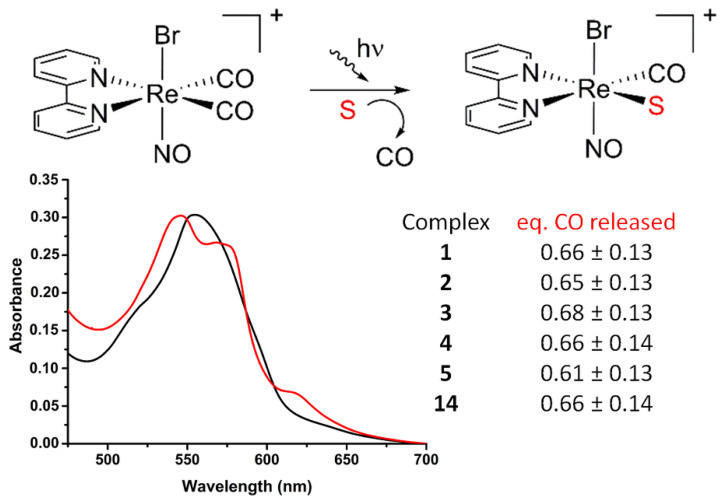
Typical UV-visible spectrum of conversion of deoxy-myoglobin (deoxy-Mb) to carbon monoxide myoglobin (MbCO) by *fac*-[Re^I^(CO)_2_(NO)(N-N)Br]BF_4_ complexes **1**–**5** and **14**. Solutions (20 μM Mb and photoCORM, 25 °C, 0.05 M phosphate buffer at pH 6.8) were exposed to a 275 nm light source (intervals of 10 min) before the conversion reached a plateau after 120 min. Prolonged exposure to the light source eventually favoured formation of metmyoglobin (MetMb), as evidenced by the peak at 620 nm. In the reaction scheme above the spectra, S = solvent molecule.

Spectrophotometric measurements of the CO release from complexes **1**–**5** and **14**, as a function of the conversion of deoxy-Mb to MbCO, revealed that dicarbonyl-mononitrosyl complexes only released the gas if exposed to a cold light source of 275–375 nm radiation. In the dark, the complexes are stable and do not elicit any change in the deoxy-Mb spectrum. A typical spectrum of the conversion of the deoxy-Mb spectrum under photolysis of **1**–**5** and **14** is given in Figure 9. The equivalents of CO released by the molecules were calculated by measuring the change of absorbance at 540 nm (ε_540_ = 15.4 mmol L^−1^ cm^−1^), as a direct measure of the concentration of MbCO. The amount of the formed MbCO is dependent on the concentration of the complex, but analysis of the saturation curve of MbCO showed, that for all tested complexes approximately 0.6–0.7 moles CO are released per mole of complex. In Figure 9, the peak at 620 nm is attributable to the formation of metmyoglobin (MetMb) after prolonged exposure to the light source [54]. The loss of CO was verified by MS experiments, whereby solutions of the complexes were irradiated under similar conditions in CH_3_CN solutions. The spectra consistently revealed that only CO is released by the species during the irradiation process and that a solvent molecule replaces the ligand.

To our knowledge, *fac*-[Re(CO)_2_(NO)(N-N)Br]^+^ complexes represent a new class of Re photoCORMs. Spontaneous and light-induced CO release of 16- and 17-electron rhenium complexes [55,56,57], and 18-electron *fac*-[Re^I^(CO)_3_(N-N)PR_3_]^+^ (where PR_3_ = phosphine or phosphite) is well known [58,59,60]. In the last five years several examples of diimine complexes bearing either σ- or π-donating ancillary ligands active towards CO photo-substitution have also been described [61,62,63,64,65]. For PR_3_ species, the strong *trans*-labilizing ability of the phosphorus donor of π-acid ligands is crucial for activating Re tricarbonyl species towards photochemical substitution of CO, and works in conjunction with the internal conversion between the ^3^MLCT and thermally accessible higher energy photoexcited ^3^LF state that is productive in terms of CO dissociation [66]. For tricarbonyl rhenium diimine complexes with π-basic ligands, mechanistic studies and picosecond time-resolved IR measurements indicate that only irradiation with higher energy photons can induce photochemical ligand substitution reactions via higher energy vibrational states rather than the lowest-lying ^3^MLCT excited or thermally accessible ^3^LF states. These higher vibrational levels are those of the ^1^MLCT state and/or higher electronic excited state(s) including the Re → CO ^1^MLCT transition. Thus, excitation by high-energy light of *fac*-[Re(CO)_3_(N-N)(L)]^n^ species (were L = π-base or σ-donor ligand), leads to vibrationally hot photoproducts, which relax within 50−100 ps, while CO ligand dissociation occurs with subpicosecond rates after excitation [67]. A similar mechanism is likely to be at play here.

### 2.7. Antimicrobial Properties of Selected Complexes

As mentioned in the introduction, this study was initiated aiming at the synthesis of structurally nearly identical higher charge congeners of active antimicrobial rhenium tricarbonyl complexes. Our previous reports on those species indicate that neutral *fac*-[Re(CO)_3_(N-N)Br] bromo complexes are inactive, whereas several cationic complexes of general formula *fac*-[Re(CO)_3_(N-N)L]^+^ (L = pyridine, py, type-ligand) show potent in vitro and in vivo activity against infection of clinically relevant bacteria (MRSA) and fungi (*Candida* species) [19,20]. We did not succeed in isolating stable *fac*-[Re(CO)_2_(NO)(N-N)L]^2+^ species, but we decided to test the antimicrobial properties of the corresponding monocationic nitrosyl complexes *fac*-[Re(CO)_2_(NO)(N-N)X]^+^ (**1**–**4** and **12**, where X = Br or BF_4_). The antimicrobial activity of **1**–**4** and **12** was determined against four Gram-negarive bacteria (*E. cloaceae*, *K. pneumoniae*, *A. baumanii*, and *P. aeruginosa*), two Gram-positive bacteria (*S. aureus* MRSA43300 and *S. aureus*) and two fungi (*C. albicans* and *C. auris*) and compared to the corresponding *fac*-[Re(CO)_3_(N-N)Br] complexes. Our data indicate that the tested *fac*-[Re(CO)_2_(NO)(N-N)X]^+^ species behave as the corresponding neutral *fac*-[Re(CO)_3_(N-N)Br] complexes and none of these complexes exhibited antimicrobial activity, with minimum inhibitory concentrations (MICs) >100 μM.

## 3. Materials and Methods

### 3.1. Reagents and Chemicals

All reagent and solvents were purchased from standard sources and used without further purification. Compound [Re(CO)_5_Br] was purchased from Sigma Aldrich, while complexes of formula *fac*-[Re^I^(CO)_3_(N-N)Br] [19], (Et_4_N)[Re^II^(CO)_2_Br_4_][36], *fac*-[Re^I^(CO)_2_(N-N)(py)Br] [37], *fac*-[Re(CO)_3_(bpy)OH] [68] and *fac*-[Re(CO)_3_(bpy)(Me_2_N-py)](CF_3_SO_3_) [46] were synthesized according to published procedures. Unless otherwise noted, solvents used in the preparation of all molecules were dry and O_2_-free.

### 3.2. Instruments and Analysis

NMR spectra were measured on a Bruker Advance III 400 MHz. The corresponding ^1^H chemical shifts are reported relative to residual solvent protons. Mass analyses were performed using a Bruker FTMS 4.7-T Apex II in positive mode. UV-Vis spectra were measured on a Jasco V730 spectrophotometer. IR spectra were recorded on a Bruker TENSOR II with the following parameters: 16 scans for background, 32 scans for sample with a resolution of 4 cm^−1^ in the 4000–600 cm^−1^ region. Single crystal diffraction data collection was performed on a Stoe IPDS2 diffractometer (CuKα1 (λ = 1.5406 Å)) equipped with a cryostat from Oxford Cryosystems. The structures were solved with the ShelXT structure solution program using Intrinsic Phasing and refined with the ShelXL refinement package using Least Squares minimization [69,70]. All crystal structures are deposited at the Cambridge Crystallographic Data Centre. CCDC numbers 2093631-2093640 and 2094070 contain the supplementary crystallographic data for this paper. These data can be obtained free of charge from the Cambridge Crystallographic Data Centre via www.ccdc.cam.ac.uk/structures (accessed on 20 July 2021).

### 3.3. Synthetic Procedures

The following general procedure was followed for the synthesis of [Re(CO)_2_(NO)(N-N)Br]BF_4_ complexes. In a glove box the corresponding *fac*-[Re(CO)_3_(N-N)Br] (typically 0.2 mmol ca. 100 mg) was dissolved in CH_2_Cl_2_ (100 mL, dry). To the resulting yellow solution, NOBF_4_ (84–115 mg, 0.5–1 mmol, 2.5–5 eq.) was added in the solid form. The reaction was stirred at room temperature for 2 days. A light off-white precipitate was filtered off and the filtrate either allowed to evaporate or layered with pentane to afford light yellow crystals, which were collected by filtration.

**[Re**(**CO**)**_2_**(**NO**)(**bpy**)**Br]**(**BF_4_**) (**1**). Yellow solid, yield 70%. ESI^+^-MS (MeOH): *m*/*z*, 507.7 [Re(CO)_2_(NO)(C_10_H_8_N_2_)Br]^+^, [M]^+^. IR (solid, cm^−1^); νCO: 2112, 2050, νNO: 1801. ^1^H-NMR (400 MHz, CD_3_CN, ppm): 7.87 (ddd, *J* = 7.70, 5.62, 1.34 Hz, 2 H) 8.48 (td, *J* = 7.98, 1.53 Hz, 2 H) 8.64 (d, *J* = 8.19 Hz, 2 H) 9.22–9.30 (m, 2 H). UV-Vis (CH_3_CN, λ nm): 312, 321. Crystals suitable for X-ray diffraction were obtained by slow evaporation of a dichloromethane solution. Elemental analysis, calcd. for C_12_H_8_B_1_Br_1_F_4_N_3_O_3_Re_1_ (%): C 24.22, H 1.36, N 7.06; found: C 24.11, H 1.29, N 7.04.

**[Re**(**CO**)**_2_**(**NO**)(**phen**)**Br]**(**BF_4_**) (**2**). Yellow solid, yield 55%. ESI^+^-MS (MeOH): *m*/*z*, 531.6 [Re(CO)_2_(NO)(C_12_H_8_N_2_)Br]^+^, [M]^+^. IR (solid, cm^−1^); νCO: 2118, 2057, νNO: 1790. ^1^H-NMR (400 MHz, DMSO-d6, ppm): 8.34 (dd, *J* = 8.31, 5.26 Hz, 2 H) 8.49 (s, 2 H) 9.25 (dd, *J* = 8.31, 1.22 Hz, 2 H) 9.89 (dd, *J* = 5.26, 1.22 Hz, 2 H). UV-Vis (CH_3_CN, λ nm): 304, 339, 375. Crystals suitable for X-ray diffraction were obtained from layering a solution of acetonitrile with diethylether/hexane (1:1). Elemental analysis, calcd. for C_14_H_8_B_1_Br_1_F_4_N_3_O_3_Re_1_ (%): C 27.16, H 1.30, N 6.79; found: C 27.61, H 1.33, N 6.66.

**[Re**(**CO**)**_2_**(**NO**)(**Et_2_N-bpy**)**Br]**(**BF_4_**) (**3**). Et_2_N-bpy = N^4^,N^4^,N^4′^,N^4′^-tetraethyl-[2,2′-bipyridine]-4,4′-diamine. Yellow solid, yield 87%. ESI^+^-MS (MeOH): *m*/*z*, 649.8 [Re(CO)_2_(NO)(C_18_H_26_N_4_)Br]^+^, [M]^+^. IR (solid, cm^−1^); νCO: 2104, 2040, νNO: 1770. ^1^H-NMR (400 MHz, CD_3_CN, ppm): 1.22–1.28 (m, 12 H) 3.64 (br. s., 8 H) 6.79 (dd, *J* = 7.03, 2.87 Hz, 2 H) 7.37 (d, *J* = 2.93 Hz, 2 H) 8.51 (d, *J* = 7.09 Hz, 2 H). UV-Vis (CH_3_CN, λ nm): 342, 357. Elemental analysis, calcd. for C_20_H_26_B_1_Br_1_F_4_N_5_O_3_Re_1_ (%): C 32.58, H 3.55, N 9.50; found: C 33.14, H 3.60, N 9.32.

**[Re**(**CO**)**_2_NO**(***t*Bu-bpy**)**Br]**(**BF_4_**) (**4**)**.***t*Bu-bpy = 4,4′-di-*tert*-butyl-2,2′-bipyridine. Yellow solid, yield 69%. ESI^+^-MS (MeOH): *m*/*z*, 619.7 [Re(CO)_2_(NO)(C_18_H_24_N_2_)Br]^+^, [M]^+^. IR (solid, cm^−1^); νCO: 2114, 2053, νNO: 1797. ^1^H NMR (400 MHz, CD_3_CN, ppm): 1.42–1.54 (m, 18 H) 7.84 (dd, *J* = 6.05, 2.02 Hz, 2 H) 8.58–8.61 (m, 2 H) 9.09–9.14 (m, 2 H). UV-Vis (CH_3_CN, λ nm): 309, 318. Elemental analysis, calcd. for C_20_H_24_B_1_Br_1_F_4_N_3_O_3_Re_1_ (%): C 33.96, H 3.42, N 5.94; found: C 34.36, H 3.47, N 5.72.

**[Re**(**CO**)**_2_NO**(**φ-phen**)**Br]**(**BF_4_**) (**5**)**.** φ-phen = 4,7-diphenyl-1,10-phenanthroline. Yellow solid, yield 61%. ESI^+^-MS (MeOH): *m*/*z*, 683.7 [Re(CO)_2_(NO)(C_24_H_16_N_2_)Br]^+^, [M]^+^. IR (solid, cm^−1^); νCO: 2114, 2052, νNO: 1795. ^1^H-NMR (400 MHz, CD_3_CN, ppm): 7.69–7.73 (m, 10 H) 8.14 (d, *J* = 5.50 Hz, 2 H) 8.26 (s, 2 H) 9.69 (d, *J* = 5.62 Hz, 2 H). UV-Vis (CH_3_CN, λ nm): 297, 336, 375. Elemental analysis, calcd. for C_26_H_16_B_1_Br_1_F_4_N_3_O_3_Re_1_ (%): C 40.49, H 2.09, N 5.45; found: C 40.89, H 2.15, N 5.13.

**[Re**(**CO**)**_2_**(**NO**)(**bpy**)**F]**(**BF_4_**) (**6**). Complex **1** (50.7 mg) was dissolved in methanol (5 mL) and the solution was heated to 55 °C. Pyridine (20.2 mg) and silver triflate (33 mg) were added in the dark. The mixture was stirred at 55 °C overnight. After filtration, the filtrate was dried under reduced pressure. The crude product was then dissolved in a minimal amount of methanol and precipitated with cold diethylether to give **6** as a white/pale yellow solid (11.4 mg, 25%) which contained traces of pyridine. ESI^+^-MS (MeOH): *m*/*z*, 447.9 [Re(CO)_2_(NO)(C_10_H_8_N_2_)F]^+^, [M]^+^. IR (solid, cm^−1^): νCO: 2113, 2051, νNO: 1779. ^1^H-NMR (400 MHz, CD_3_CN, ppm): 8.65 (ddd, *J* = 4.7, 1.7, 1.0 Hz, 2 H), 8.41 (dt, *J* = 8.0, 1.0 Hz, 2 H), 7.89 (td, *J* = 7.8, 1.8 Hz, 2 H), 7.38 ppm (ddd, *J* = 7.5, 4.8, 1.2 Hz, 2 H). UV-Vis (DMF, nm): 307, 316. Crystals suitable for X-ray diffraction were obtained by layering diethylether on a methanol solution of the compound.

**[Re**(**CO**)**_2_**(**NO**)(**Et_2_N-bpy**)**F]**(**BF_4_**) (**7**). Complex **3** (80.0 mg) was dissolved in 5 mL of methanol. The solution was then heated to 55 °C and pyridine (25.8 mg, 3 eq.) was added followed by the addition of silver triflate (41.8 mg, 1.5 eq.) in the dark. The reaction mixture was stirred over night at 55 °C, filtered and the solvent evaporated. The crude product was then dissolved in a minimal amount of methanol and precipitated with cold diethylether to give **7** as a white/beige solid (21.0 mg, ca. 29%) which contained traces of pyridine. ESI^+^-MS (MeOH): *m*/*z*, 589.67 [Re(CO)_2_(NO)(C_18_H_26_N_4_)F]^+^, [M]^+^. IR (solid, cm^−1^): νCO: 2106, 2033, νNO: 1784. ^1^H-NMR (400 MHz, CD_3_CN, ppm): 1.25 (t, *J* = 7.15 Hz, 12 H) 3.63 (d, *J* = 7.09 Hz, 8 H) 6.77 (dd, *J* = 7.09, 2.93 Hz, 2 H) 7.35 (d, *J* = 2.81 Hz, 2 H) 7.99 (t, *J* = 7.09 Hz, 2 H) 8.46–8.60 (m, 3 H) 8.69 (d, *J* = 5.26 Hz, 2 H). UV-Vis (DMF, nm): 342, 352. Crystals suitable for X-ray diffraction were obtained by layering diethylether on a methanol solution of the compound.

**[Re**(**CO**)**_2_**(**py**)(**bpy**)**Br]** (**8**). This complex was prepared by following a previously published procedure for similar species [37]. Brown solid, yield 80%. IR (solid, cm^−1^); νCO: 1864, 1781. ^1^H-NMR (400 MHz, CD_2_Cl_2_, ppm): 6.83 (dd, *J* = 7.52, 6.66 Hz, 2 H) 7.37–7.49 (m, 3 H) 7.89 (td, *J* = 7.89, 1.47 Hz, 2 H) 8.09 (d, *J* = 8.07 Hz, 2 H) 8.37 (dd, *J* = 6.60, 1.47 Hz, 2 H) 9.16 (d, *J* = 5.38 Hz, 2 H). UV-Vis (DMF, λ nm): 393, 308. Crystals suitable for X-ray diffraction were obtained by layering pentane on a DCM solution of the compound.

**[Re**(**CO**)**_2_NO**(**bpy**)(**Me_2_N-py**)**]**(**BF_4_**)**_2_** (**9**). In a glove box *fac*-[Re(CO)_3_(bpy)(Me_2_N-py)](CF_3_SO_3_) [46] (30 mg, 0.043 mmol, where Me_2_N-py = *N*,*N-*dimethylpyridin-4-amine) was dissolved in CH_2_Cl_2_ (30 mL, dry) and to the resulting yellow solution, NOBF_4_ (10 mg, 2 eq.) was added in the solid form. The reaction was stirred at room temperature for 2 days. A light yellow solid appeared. It was filtered off, washed with cold CH_2_Cl_2_ and dried in vacuo. Attempts to purify the salt led to the decomposition of the product. Yield 62%. IR (solid, νCO cm^−1^): 2129, 2070, νNO: 1830. ^1^H NMR (400 MHz, CD_3_CN, ppm): 9.24–9.31 (m, 2H), 8.67–8.73 (m, 2H), 8.52–8.61 (m, 2H), 7.98–8.02 (m, 2H), 7.91–7.97 (m, 2H), 6.87 (d, J = 6.85 Hz, 2H), 3.19–3.22 (m, 6H).

The *fac*-[Re(CO)_3_(bpy)] carboxylato complexes were prepared by adaptation of a published procedure [71]. Briefly, *fac*-[Re(CO)_3_(bpy)Br] (100 mg, 0.2 mmol) and the appropriate carboxylic acid ligand were dissolved in anhydrous degassed tetrahydrofuran (30 mL). The reaction mixture was stirred for 5 min, then trimethylamine (33 μL, 24 mg, 0.24 mmol) and AgCF_3_SO_3_ (50 mg, 0.2 mmol) were added, and the mixture stirred at 70 °C for 18 h. The mixture was filtered, solvent evaporated to dryness and the residue purified by column chromatography on deactivated alumina with DCM as the eluent. Analytically pure products were obtained after recrystallization from dichloromethane/n-hexane mixtures.

**[Re**(**CO**)**_3_**(**bpy**)(**O_2_CBz**)**]** (**10**). O_2_CBz = benzoate**.** Yellow solid, yield 59%. ESI^+^-MS (MeOH): *m*/*z*, 571 [Re(CO)_3_(C_10_H_8_N_2_)(C_7_H_5_O_2_)] + Na^+^, [M + Na]^+^. IR (solid, cm^−1^); νCO: 2012, 1904, 1866. ^1^H-NMR (400 MHz, CD_3_CN, ppm): 9.08–9.15 (m, 2 H) 8.41 (d, *J* = 8.19 Hz, 2 H) 8.20 (td, *J* = 7.92, 1.53 Hz, 2 H) 7.58–7.67 (m, 2 H) 7.38 (dd, *J* = 8.01, 1.28 Hz, 2 H) 7.21–7.29 (m, 1 H) 7.08–7.17 (m, 2 H). UV-Vis (DMF, λ nm): 369, 317, 293. Crystals suitable for X-ray diffraction were obtained from layering a solution of tetrahydrofuran with pentane.

**[Re**(**CO**)**_3_**(**bpy**)(**O_2_CPh**)**]** (**11**). O_2_CPh = 4-bromobenzenecarboxylate**.** Yellow solid, yield 59%. ESI^+^-MS (MeOH): *m*/*z*, 663 [Re(CO)_3_(C_10_H_8_N_2_)(C_8_H_6_O_2_Br)] + Na^+^, [M + Na]^+^. IR (solid, cm^−1^); νCO: 2011,1874. ^1^H-NMR (400 MHz, CD_3_CN, ppm): 8.93 (dt, *J* = 4.71, 0.70 Hz, 2 H) 8.18–8.23 (m, 2 H) 8.11–8.17 (m, 2 H) 7.54 (ddd, *J* = 7.34, 5.62, 1.47 Hz, 2 H) 7.01–7.13 (m, 2 H) 6.49 (d, *J* = 8.56 Hz, 2 H) 2.97–3.00 (m, 2 H). UV-Vis (DMF, λ nm): 368 319, 293. Crystals suitable for X-ray diffraction were obtained from layering a solution of tetrahydrofuran with pentane.

**[Re**(**CO**)**_2_NO**(**bpy**)**BF_4_]**(**BF_4_**) (**12**)**.** Compound **10** or **11** (20–25 mg) was dissolved in dry DCM (20 mL). NOBF_4_ (1.5 eq.) was added in solid form to the resulting solution. The reaction mixture was stirred for 24h at room temperature in dark. A white solid appeared, and then the solvent was decanted from the mixture. The residue was dissolved in DCM and water (1:1). The two phases were separated, and the aqueous phase was washed with DCM. The aqueous phase was concentrated under vacuum. The crude product was crystalized by slow evaporation of a CHCl_3_: acetone (1:1) solution to yield crystals of the pure product **12**. White solid, yield 55%. ESI^+^-MS (MeOH): m/z, 515.5 [Re(CO)_2_(NO)(C_10_H_8_N_2_)BF_4_]^+^. IR (solid, cm^−1^); νCO: 2127, 2069, νNO: 1820. ^1^H NMR (400 MHz, CD_3_CN, ppm): 9.24–9.27 (dd, 2H), 8.62–8.65 (dd, 2H), 8.45–8.50 (t, 2H), 7.85–7.89 (td, 2H). Crystals suitable for X-ray diffraction were obtained by slow evaporation of an acetone solution. Elemental analysis, calcd. for C_12_H_8_B_2_F_8_N_3_O_3_Re_1_ (%): C 23.94, H 1.34, N 6.98; found: C 24.43, H 1.35, N 6.69.

**[Re**(**CO**)**_2_NO**(**6Me-bpy**)**Br]**(**BF_4_**) (**14**)**.** Prepared according to general procedure for **1**–**5**. 6Me-bpy = 6-methyl-2,2′-bipyridine. Yellow solid, yield 67%. ESI^+^-MS (MeOH): *m*/*z*, 521.6 [Re(CO)_2_(NO)(C_11_H_10_N_2_)Br]^+^, [M]^+^. IR (solid, cm^−1^); νCO: 2114, 2050, νNO: 1796. ^1^H-NMR (400 MHz, DMSO-d6, ppm): 3.13 (s, 3 H) 7.98 (ddd, *J* = 7.40, 5.87, 1.16 Hz, 1 H) 8.02–8.07 (m, 1 H) 8.47 (t, *J* = 7.95 Hz, 1 H) 8.59 (td, *J* = 7.95, 1.47 Hz, 1 H) 8.82 (d, *J* = 7.58 Hz, 1 H) 8.94 (d, *J* = 8.31 Hz, 1 H) 9.50 (dd, *J* = 5.69, 0.92 Hz, 1 H). UV-Vis (CH_3_CN, λ nm): 323, 332. Crystals suitable for X-ray diffraction were obtained by vapor diffusion of pentane into a dichloromethane solution.

### 3.4. Detection of CO Release Using the Myoglobin Assay

The photorelease of CO from **1**-**5** and **13** was assessed spectrophotometrically by measuring the conversion of deoxymyoglobin (Mb) to carbonmonoxy myoglobin (MbCO) as previously reported [72]. A small aliquot of a freshly prepared solution of the selected complex (in DMSO) was added to 1 mL of the Mb solution in phosphate buffer (0.05 M) prepared at pH 6.8. Final concentrations: 20 μM for Re complex and Mb. Mb spectra were recorded after each photoirradiation (5–10 min) at 375nm at 25 °C. The methanol or DMSO content of the solution never exceeded 0.5%. The amount of MbCO formed was determined by measuring the absorbance at 540 nm (extinction coefficient ε = 15.4 M cm^−1^). The MbCO concentration was plotted over time and directly related to the equivalents of CO released from the compounds. Control experiments were run under identical conditions but without light or the addition of the metal complexes.

### 3.5. Strains and Culture Conditions

The antimicrobial activity of selected [Re(CO)_2_(NO)(N-N)X]BF_4_ was evaluated against 8 different microorganisms including four Gram-negative bacteria (*Enterobacter cloaceae* ATCC 3047, *Klebsiella pneumoniae* ATCC 13803, *Acinetobacter baumanii* ATCC 19606, *Pseudomonas aeruginosa* PAO1 NCTC10332), two Gram-positive bacteria (*Staphylococcus aureus* MRSA43300 (methicillin-resistant) and *S. aureus* ATCC25923 (methicillin-sensitive)) and two fungi (*Candida albicans* SC5314) and *C. auris* (a clinical strain)). All reference strains were obtained from the American Type Culture Collection (ATCC) and the National Collection of Type Cultures (NCTC), while the clinical *C. auris* strain 7 was kindly provided by Dr Aleksandra Barac (University Clinical Center of Serbia) and Prof. Cornelia Lass-Floerl (University of Innsbruck). Prior to each experiment, frozen stocks in 20% glycerol at −80 °C were thawed and inoculated onto solid Yeast-Potato Dextose (YPD) plates (fungi) or Lauria (LA) agar plates (bacteria), and cultured at 37 °C for 24–48 h.

### 3.6. In Vitro Antimicrobial Activity Determination

Antimicrobial activity was addressed by determining the minimum inhibitory concentration (MIC) of the tested complexes according to the standard broth microdilution assays, recommended by CLSI (the Clinical and Laboratory Standards Institute; M07-A10. CLSI) and EUCAST (European Committee on Antimicrobial Susceptibility Testing; EUCAST antifungal MIC method for yeasts, v 7.3.1). The test strains grown in YPD (fungi) and LA (bacteria) were diluted in RPMI 1640 medium with 2% glucose (Gibco) and Luria-Bertani broth (Biolife Italiana S.r.l., Milano, Italy) to give the concentration of 1 × 105 CFU/mL cells (for fungi) and 5 × 105 CFU/mL (for bacteria), respectively. The MIC assay was performed in 96-well microtiter plates (Sarstedt, Germany) by making serial twofold dilutions of the tested substances in appropriate liquid media to give the volume of 100 µL. The media solution with microorganisms was dispensed to each well to make the final volume of 200 µL. All complexes were tested in the concentrations range from 100 to 3.13 µM. After incubation at 37 °C for 18–24 h without shaking, the growth of tested microorganisms was determined measuring absorbance at 530 nm (fungi) and 600 nm (bacteria) using a Tecan Infinite 200 Pro multiplate reader (Tecan Group Ltd., Männedorf, Switzerland). The negative control (media only) and positive control (only microorganisms) on the same plate were used as references to determine the growth inhibition. Samples with inhibition values above 90% were classified as active agents.

## 4. Conclusions

In this contribution, we have described an efficient direct nitrosylation of α-diimine rhenium tricarbonyl complexes widely applicable to different supporting N-N ligands and studied the substitution chemistry of the resulting species. We showed that *fac*-[Re^I^(CO)_3_(N-N)Br] complexes react efficiently with NOBF_4_ to yield the corresponding dicarbonyl-mononitrosyl species. The resulting compounds are structurally nearly identical higher charge congeners of tricarbonyls. Although the rhenium ion is formally described as having oxidation state +1, we found that the substitution chemistry of metal ion in *fac*-[Re(CO)_2_(NO)(N-N)X]^+^ is closer to that of a harder Re acid, probably due to the stronger π-acceptor properties of NO^+^ as compared to those of CO. In comparison to tricarbonyl species, the higher apparent formal oxidation state of the metal ion in dicarbonyl-mononitrosyl complexes, implies the greater depletion of electron density from the same and, consequently, lower π-back Re-CO bonding. A fundamental consequence is that *fac*-[Re(CO)_2_(NO)(N-N)X]^+^ complexes can be photo-activated towards CO release and represent a new class of photoCORMs, releasing ca. 1 equivalent of CO when photo-irradiated with UV light. Preliminary antimicrobial tests of selected *fac*-[Re(CO)_2_(NO)(N-N)X]^+^ complexes indicate that the nitrosyl species behave as the corresponding neutral *fac*-[Re(CO)_3_(N-N)Br] complexes, showing no antimicrobial activity, with minimum inhibitory concentrations (MICs) >100 μM.

## Figures and Tables

**Table 1:** Physical properties of *fac*-[Re^I^(CO)_2_(NO)(N-N)X]^+^ complexes.

Complex	ν(CO) [cm^−1^] ^a^	ν(NO) [cm^−1^] ^a^	λ_max_ [nm (M^−1^cm^−1^)] ^b^
**1**	2112, 2050	1801	312, 321
**2**	2118, 2057	1790	304, 339, 375
**3**	2104, 2040	1770	342, 357
**4**	2114, 2053	1797	309, 318
**5**	2114, 2052	1795	297, 336, 375
**6**	2113, 2051	1779	307, 316
**7**	2106, 2033	1784	342, 352
**12**	2127, 2069	1820	320, 330
**14**	2114, 2050	1796	323, 332

^a^ Solid state pure product. ^b^ in DMF.

## Data Availability

The data presented in this study are available on request from the corresponding author.

## Supplementary Materials

Figure S1: 400 MHz ^1^H-NMR of [Re(CO)_2_(NO)(bpy)Br](BF_4_) (**1**), Figure S2: 400 MHz ^1^H NMR spectrum of the [Re(CO)_2_(NO)(phen)Br](BF_4_) (**2**), Figure S3: 400 MHz ^1^H NMR spectrum of the [Re(CO)_2_(NO)(Et_2_N-bpy)Br](BF_4_) (**3**), Figure S4: 400 MHz ^1^H NMR spectrum of [Re(CO)_2_NO(*t*Bu-bpy)Br](BF_4_) (**4**), Figure S5: 400 MHz ^1^H NMR spectrum of [Re(CO)_2_NO(ϕ-phen)Br](BF_4_) (**5**), Figure S6: 400 MHz ^1^H NMR spectrum of [[Re(CO)_2_(NO)(Et_2_N-bpy)F](BF_4_) (**7**), Figure S7: 400 MHz ^1^H-NMR of [Re(CO)_3_(bpy)(O_2_CBz)] (**10**), Figure S8: 400 MHz ^1^H-NMR of [Re(CO)_3_(bpy)(O_2_CPh)] (**11**), Figure S9: 400 MHz ^1^H NMR spectrum of [Re(CO)_2_NO(bpy)BF_4_](BF_4_) (**12**), Figure S10: 400 MHz ^1^H-NMR of [Re(CO)_2_NO(6-Me-bpy)Br](BF_4_) (**14**), Figure S11: 400 MHz ^1^H NMR spectrum of [Re(CO)_2_NO(bpy)(Me2N-py)](BF_4_)_2_ (**9**), Figure S12: Comparison of the ^1^H NMR spectra (400 MHz) of (top to bottom) *N*,*N*-dimethylpyridin-4-amine (Me2N-py), *fac*-[Re(CO)_3_(bpy)(Me2N-py)](CF_3_SO_3_) (**B**) and [Re(CO)_2_NO(bpy)(Me2N-py)](BF_4_)_2_ (**9**), Figure S13: IR spectrum of [Re(CO)_2_(NO)(bpy)Br](BF_4_) (**1**), Figure S14: IR spectrum of [Re(CO)_2_NO(phen)Br](BF_4_) (**2**), Figure S15: IR spectrum of [Re(CO)_2_NO(Et_2_N-bpy)Br](BF_4_) (**3**), Figure S16: IR spectrum of [Re(CO)_2_NO(*t*Bu-bpy)Br](BF_4_) (**4**), Figure S17: IR spectrum of [Re(CO)_2_NO(ϕ-phen)Br](BF_4_) (**5**), Figure S18: IR spectrum of [Re(CO)_2_NO(bpy)F](BF_4_) (**6**), Figure S19: IR spectrum of [Re(CO)_2_NO(Et_2_N-bpy)F](BF_4_) (**7**), Figure S20: IR spectrum of [Re(CO)_2_NO(bpy)(Me2N-py)](BF_4_)_2_ (**9**), Figure S21: IR spectrum of [Re(CO)_3_(bpy)(O_2_CBz)] (**10**), Figure S22: IR spectrum of [Re(CO)_3_(bpy)(O_2_CPh)] (**11**), Figure S23: IR spectrum of [Re(CO)_2_NO(bpy)BF_4_](BF_4_) (**12**), Figure S24: IR spectrum of [Re(CO)_2_NO(6-Me-bpy)Br](BF_4_) (**14**), Figure S25: UV-Vis spectrum of [Re(CO)_2_NO(bpy)Br](BF_4_) (**1**) in acetonitrile, Figure S26: UV-Vis spectrum of [Re(CO)_2_NO(phen)Br](BF_4_) (**2**) in DMF, Figure S27: UV-Vis spectrum of [Re(CO)_2_NO(Et_2_N-bpy)Br](BF_4_) (**3**) in acetonitrile, Figure S28: UV-Vis spectrum of [Re(CO)_2_NO(*t*Bu-bpy)Br](BF_4_) (**4**) in DMF, Figure S29: UV-Vis spectrum of [Re(CO)_2_NO(ϕ-phen)Br](BF_4_) (**5**) in acetonitrile, Figure S30: UV-Vis spectrum of [Re(CO)_2_NO(6-Me-bpy)Br](BF_4_) (**14**) in acetonitrile, Figure S31: UV-Vis spectrum of [Re(CO)_2_(NO)(Et_2_N-bpy)F](BF_4_) (**7**) in acetonitrile, Figure S32: UV-Vis spectrum of [Re(CO)_3_(bpy)(O_2_CBz)] (**10**) in DMF, Figure S33: UV-Vis spectrum of [Re(CO)_3_(bpy)(O_2_CPh)] (**11**) in DMF, Figure S34: UV-Vis spectrum of [Re(CO)_2_NO(bpy)BF_4_](BF_4_) (**12**) in DMF, Figure S35: UV-Vis spectrum of [Re(CO)_2_NO(bpy)(Me_2_N-py)](BF_4_)_2_ (**9**) in acetonitrile, Figure S36: UV-Vis spectrum of [Re(CO)_2_(NO)(bpy)F](BF_4_) (**6**) in DMF, Figure S37: Emission spectra of selected nitrosyl Re complexes. Top: λ_ex_ = 318; bottom λ_ex_ = 350, Table S1: Crystal data and structure refinement for **1**, **2**, **6**–**8**, **10**–**14,** and fully oxidized complex **14**.

## Sample Availability

Not available.
