# Supplementary material for: Efficient Direct Nitrosylation of α-Diimine Rhenium Tricarbonyl Complexes to Structurally Nearly Identical Higher Charge Congeners Activable towards Photo-CO Release
Source: Molecules. 2021 Aug 31;26(17):5302. doi: 10.3390/molecules26175302 (PMC8434269; doi:10.3390/molecules26175302)
Supplement: Supplementary file 1 [file molecules-26-05302-s001.zip › molecules-1327613-supplementary.pdf]

SUPPORTING INFORMATION FOR

Efficient Direct Nitrosylation of α-Diimine Rhenium Tricarbonyl Complexes to Structurally Nearly Identical Higher Charge Congeners Activable towards Photo-CO Release

Sara Nasiri Sovari [1,†], Isabelle Kolly [1,†], Kevin Schindler [1], Youri Cortat [1], Shing-Chi Liu [1], Aurelien Crochet [1], Aleksandar Pavic [2] and Fabio Zobi [1,*]

[1] Department of Chemistry, Fribourg University, Chemin Du Musée 9, 1700 Fribourg, Switzerland;
sara.nasirisovari@unifr.ch (S.N.S.); isabelle.kolly@unifr.ch (I.K.);
kevin.schindler@unifr.ch (K.S.);
youri.cortat@unifr.ch (Y.C.); shing-chi.liu@unifr.ch (S.-C.L.); aurelien.crochet@unifr.ch (A.C.)
[2] Institute of Molecular Genetics and Genetic Engineering, University of Belgrade, Vojvode Stepe 444a,
11042 Belgrade, Serbia; sasapavic@imgge.bg.ac.rs
* Correspondence: fabio.zobi@unifr.ch
† These authors contributed equally to this paper

Table of contents

^{1}H-NMR spectra of complexes **1-14** – Figures S1-S12 – page 2-7

IR spectra of complexes **1-14** – Figures S13-S24 – page 8-13

UV-Vis spectra of complexes **1-14** – Figures S25-S36 – page 14-15

Emission spectra of selected nitrosyl Re complexes – Figures S37 – page 16

Table S1 Crystal data and structure refinement for complexes – page 17

NMR spectra

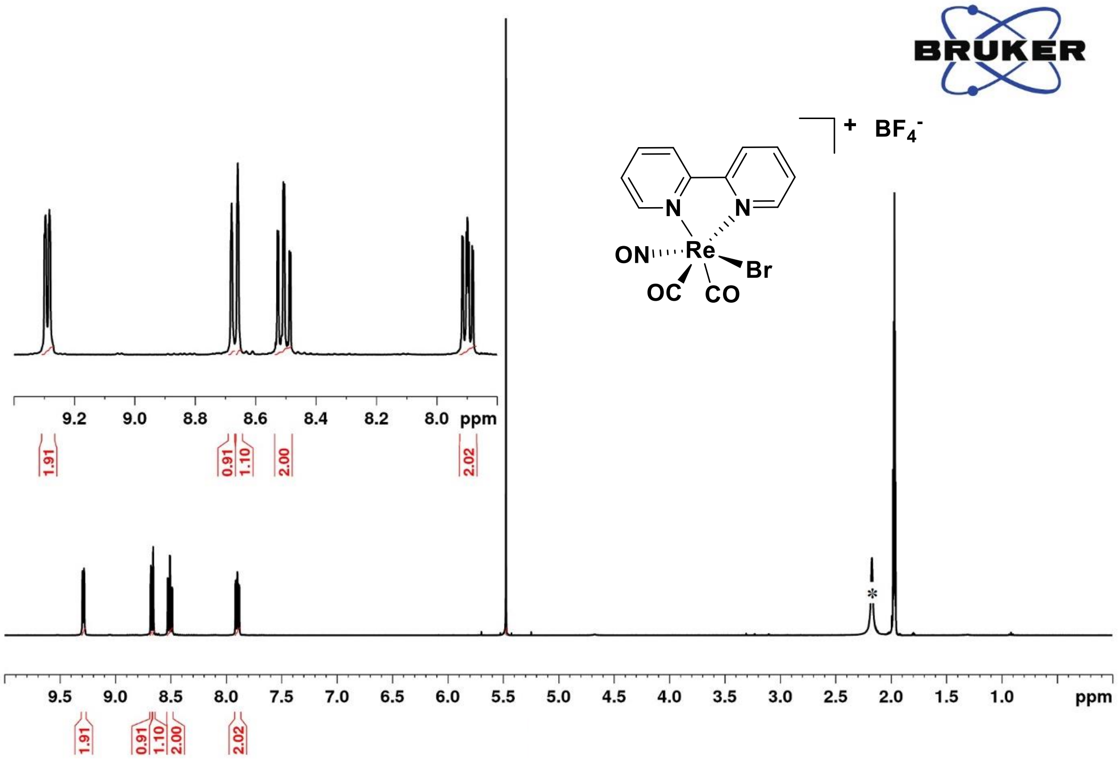

Figure S1. 400 MHz ^{1}H-NMR of [Re(CO)$_2$(NO)(bpy)Br](BF$_4$) (**1**) (in Acetonitrile, ✻ = solvent residual peak).

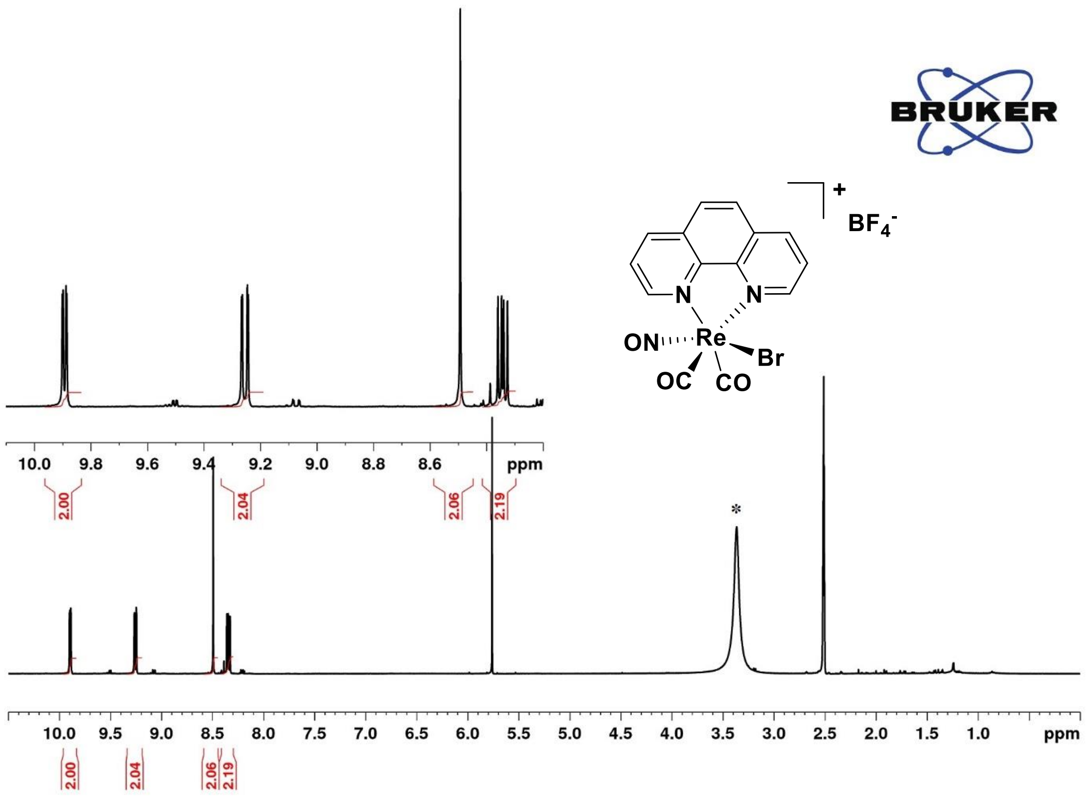

Figure S2. 400 MHz ^{1}H NMR spectrum of the [Re(CO)$_2$(NO)(phen)Br](BF$_4$) (**2**) (in DMSO-d$_6$, ✻ = solvent residual peak).

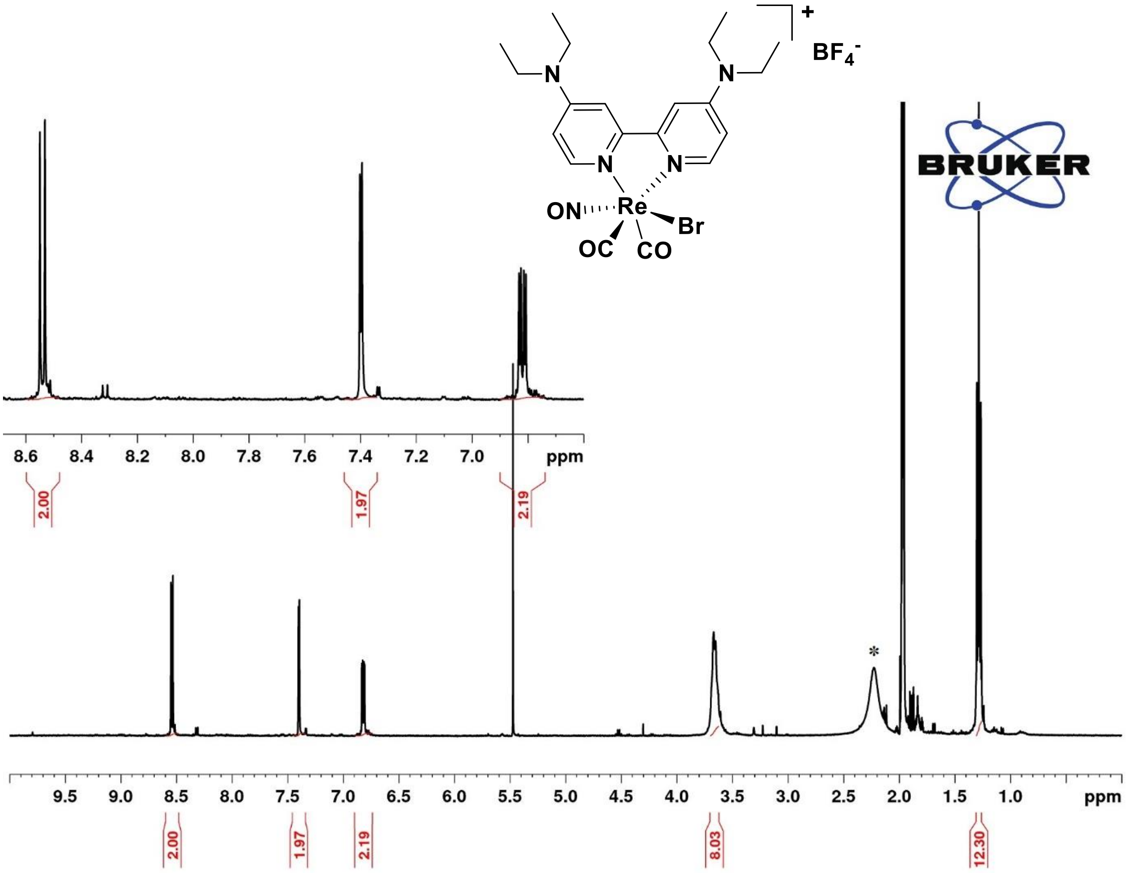

Figure S3. 400 MHz ^{1}H NMR spectrum of the [Re(CO)$_2$(NO)(Et$_2$N-bpy)Br](BF$_4$) (**3**) (in Acetonitrile, ✻= solvent residual peak).

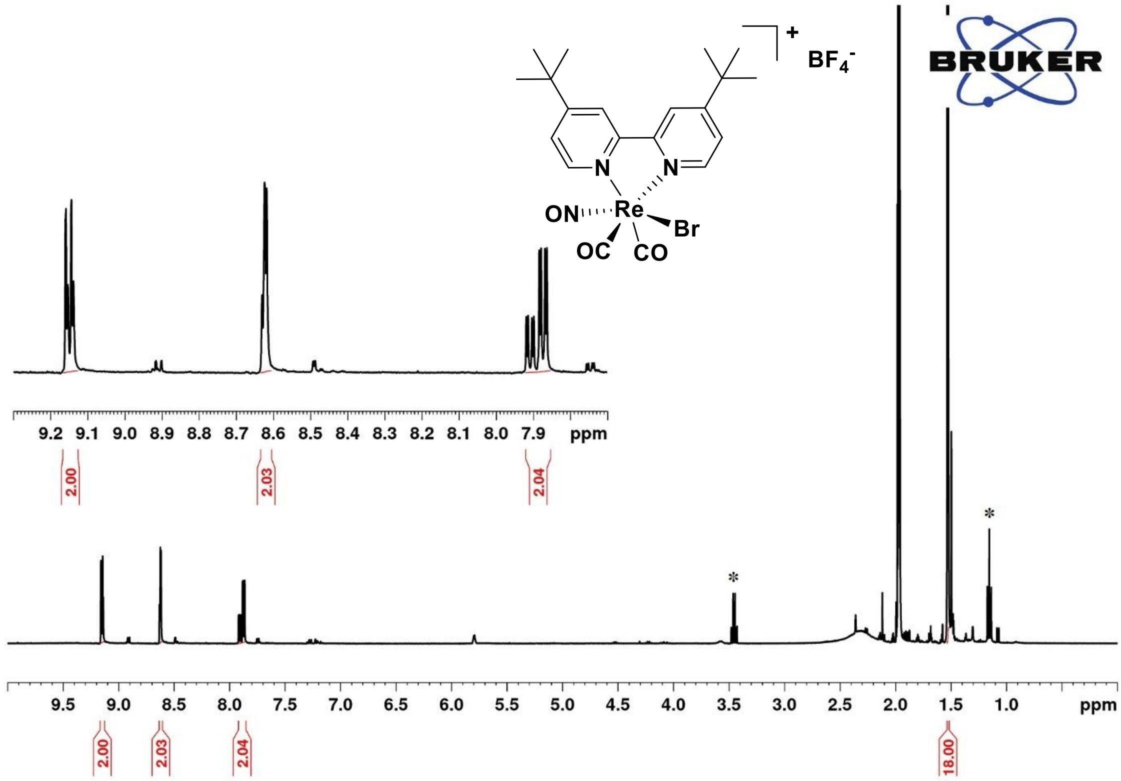

Figure S4. 400 MHz ^{1}H NMR spectrum of [Re(CO)$_2$NO(tBu-bpy)Br](BF$_4$) (**4**) (in Acetonitrile, ✻= solvent residual peak).

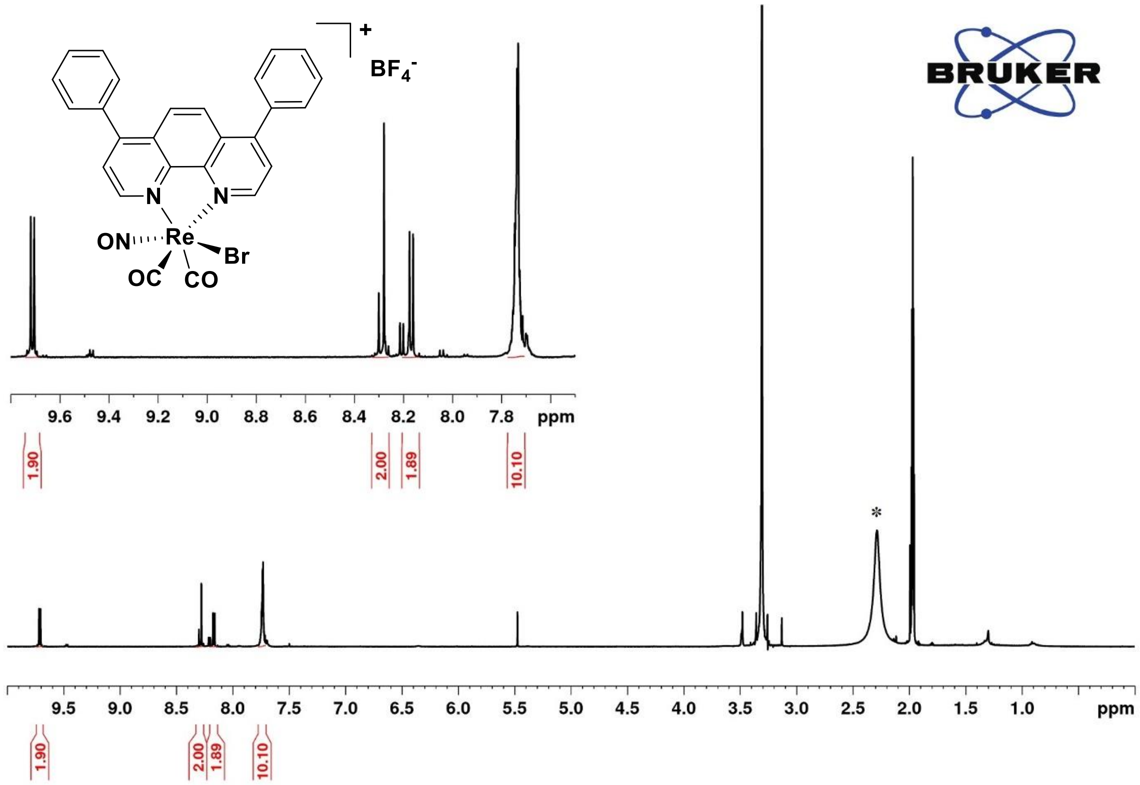

Figure S5. 400 MHz ^{1}H NMR spectrum of [Re(CO)$_2$NO(φ-phen)Br](BF$_4$) (**5**) (in Acetobitrile, ✻ = solvent residual peak).

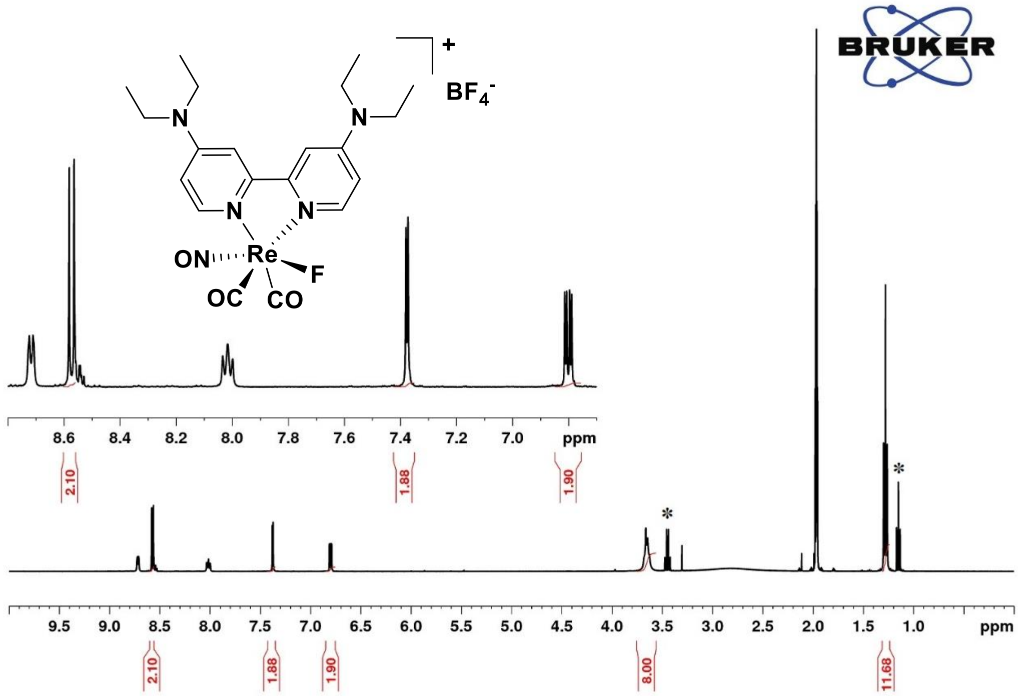

Figure S6. 400 MHz ^{1}H NMR spectrum of [[Re(CO)$_2$(NO)(Et$_2$N-bpy)F](BF$_4$) (**7**) (in Acetonitrile, ✻ = solvent residual peak).

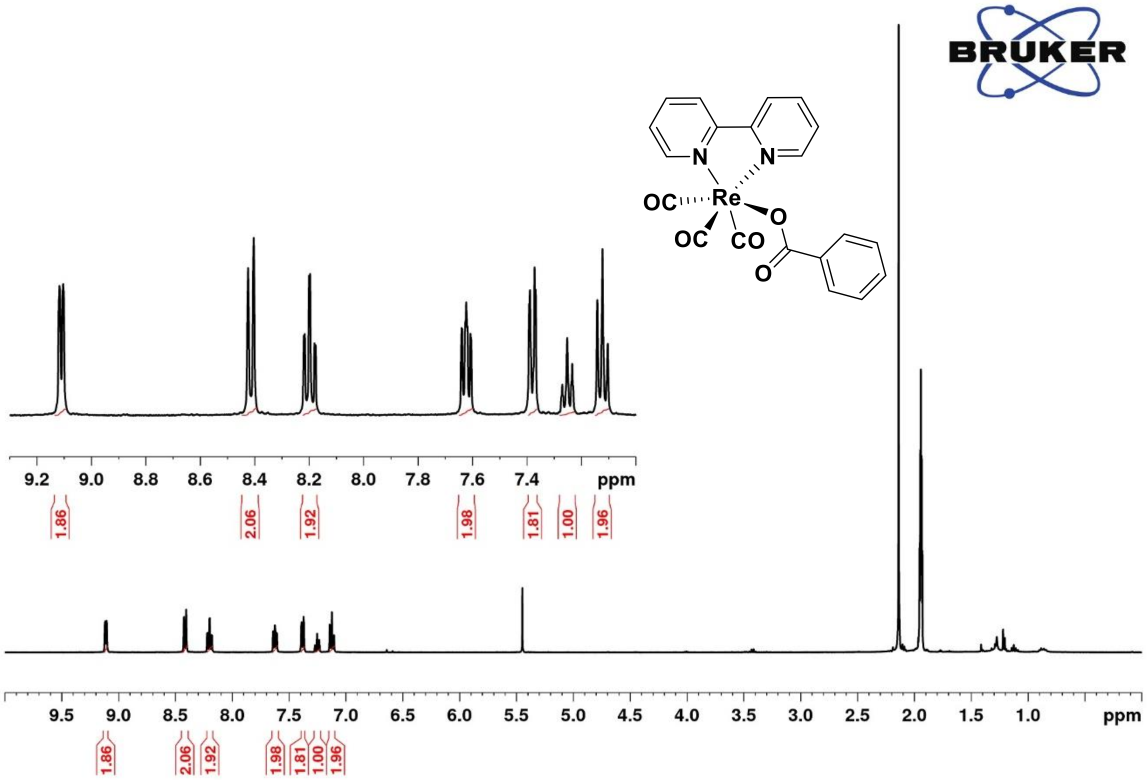

Figure S7. 400 MHz ^{1}H-NMR of [Re(CO)$_3$(bpy)(O$_2$CBz)] (**10**) (in Acetonitrile).

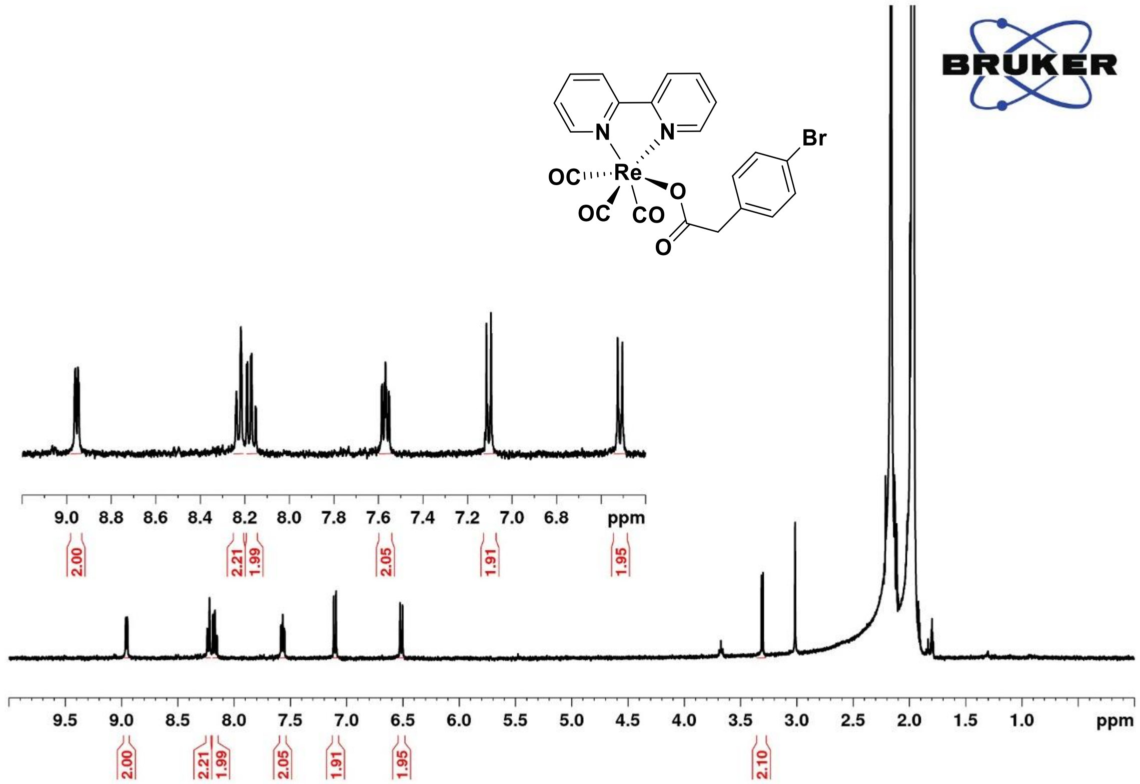

Figure S8. 400 MHz ^{1}H-NMR of [Re(CO)$_3$(bpy)(O$_2$CPh)] **(11)** (in Acetonitrile).

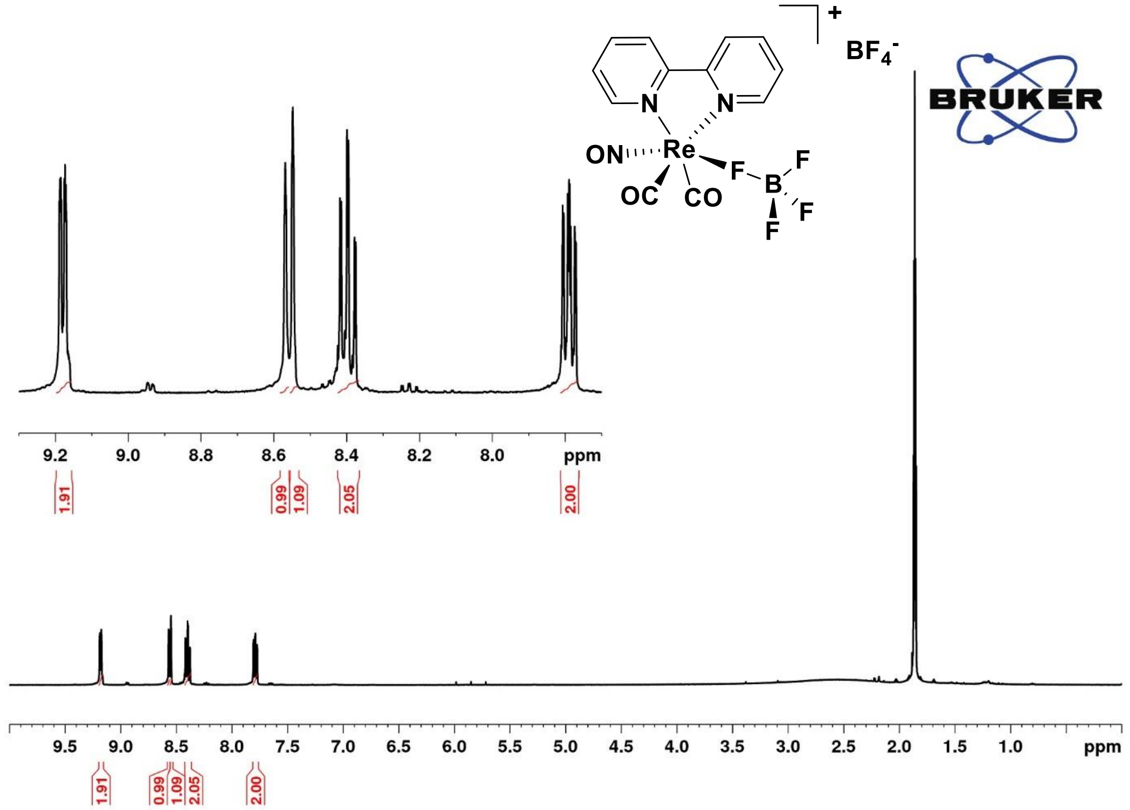

Figure S9. 400 MHz ^{1}H NMR spectrum of $[Re(CO)_2NO(bpy)BF_4](BF_4)$ **(12)** (in Acetonitrile).

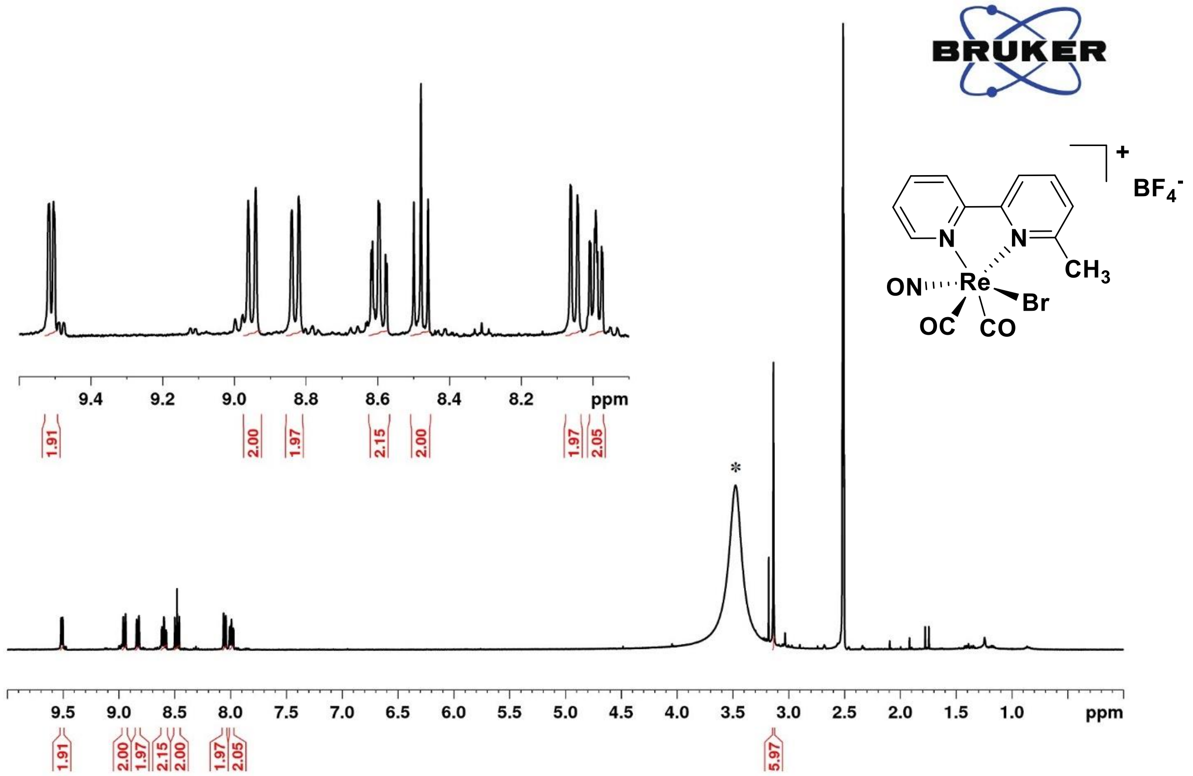

Figure S10. 400 MHz ^{1}H-NMR of $[Re(CO)_2NO(6\text{-}Me\text{-}bpy)Br](BF_4)$ (**14**) (in Acetonitrile, ✻ = solvent residual peak).

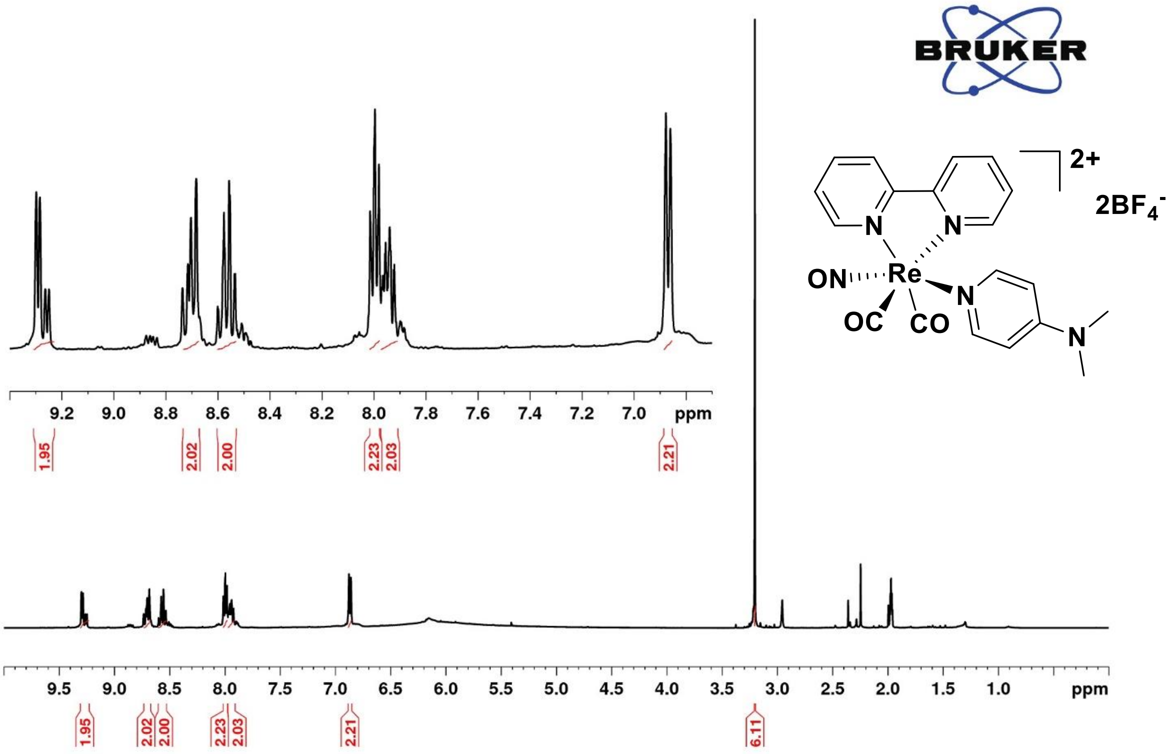

Figure S11. 400 MHz ^{1}H NMR spectrum of [Re(CO)$_2$NO(bpy)(Me2N-py)](BF$_4$)$_2$ (**9**) (in Acetonitrile).

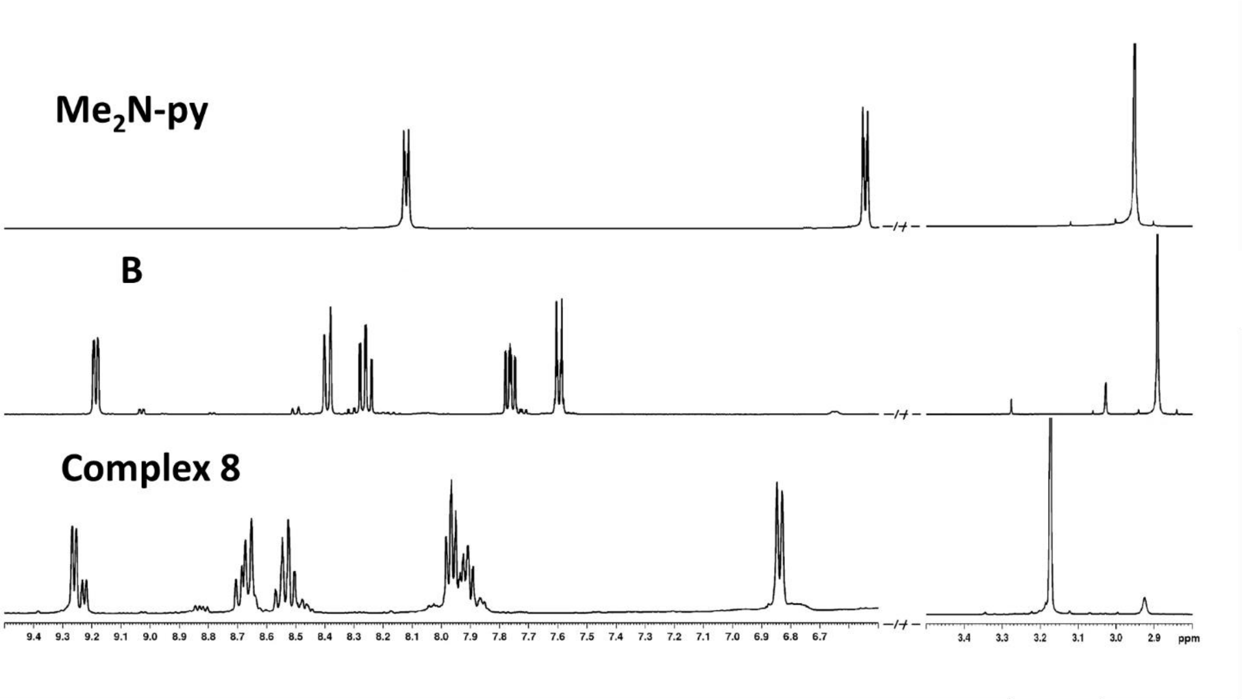

Figure S12. Comparison of the ^{1}H NMR spectra (400 MHz) of (top to bottom) *N,N*-dimethylpyridin-4-amine (Me2N-py), *fac*-[Re(CO)$_3$(bpy)(Me2N-py)](CF$_3$SO$_3$) (**B**) and [Re(CO)$_2$NO(bpy)(Me2N-py)](BF$_4$)$_2$ (**9**) (in Acetonitrile).

IR Spectra

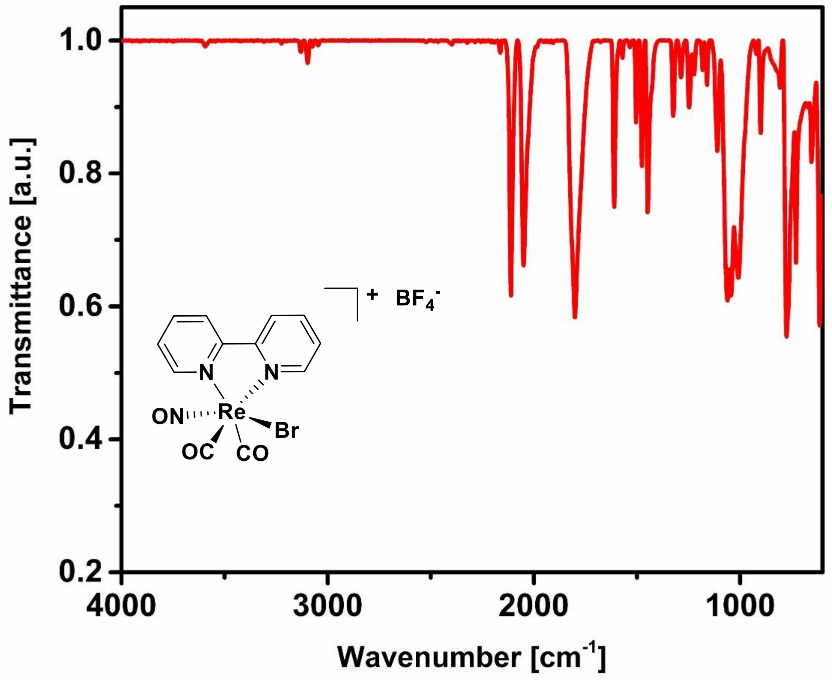

Figure S13. IR spectrum of [Re(CO)$_2$(NO)(bpy)Br](BF$_4$) (**1**).

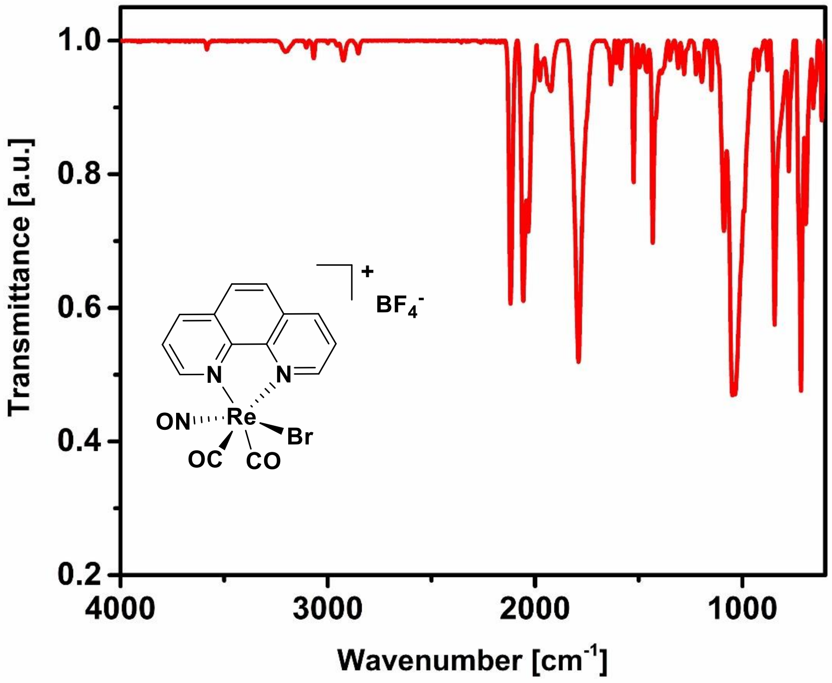

Figure S14. IR spectrum of [Re(CO)$_2$NO(phen)Br](BF$_4$) (**2**).

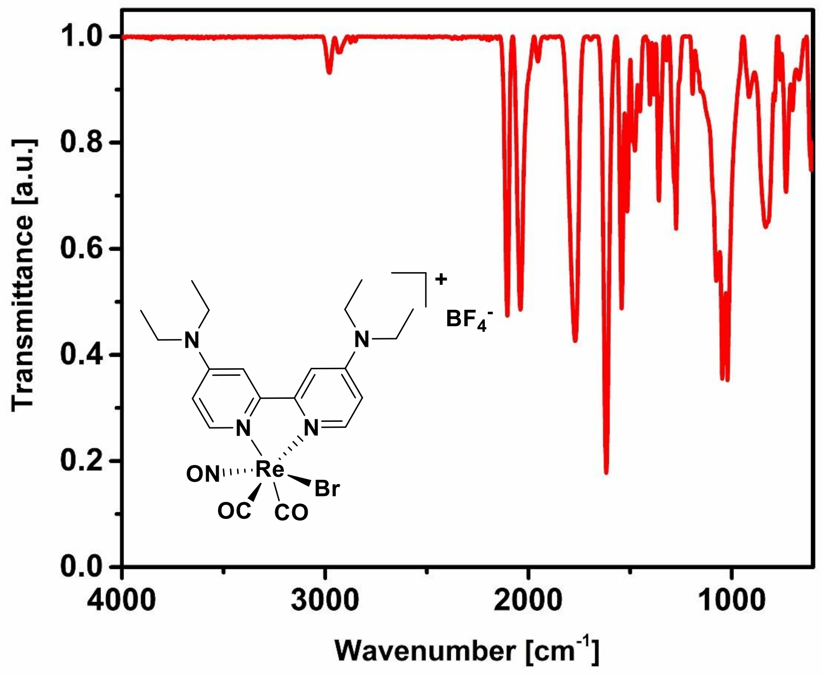

Figure S15. IR spectrum of [Re(CO)$_2$NO(Et$_2$N-bpy)Br](BF$_4$) (**3**).

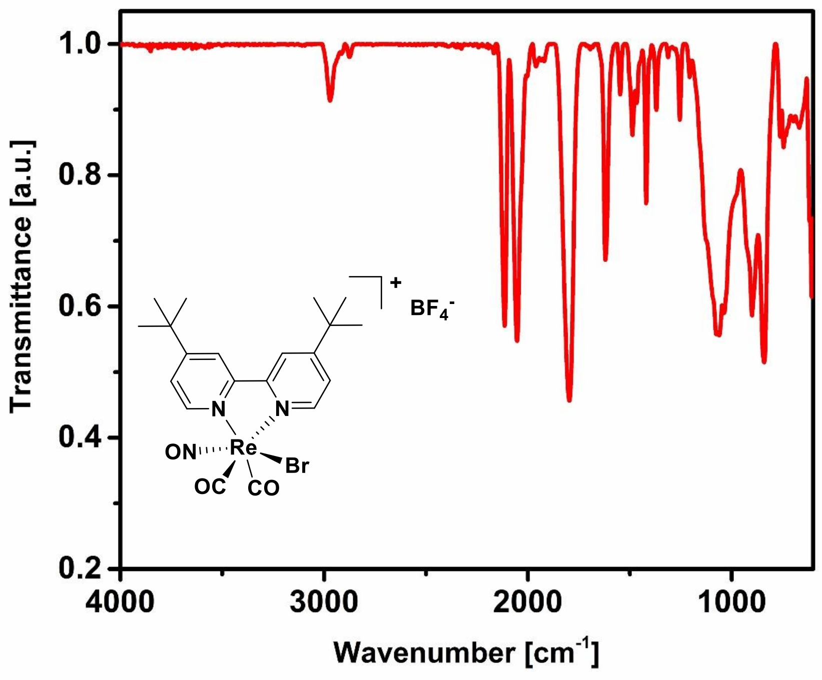

Figure S16. IR spectrum of [Re(CO)$_2$NO(tBu-bpy)Br](BF$_4$) (**4**).

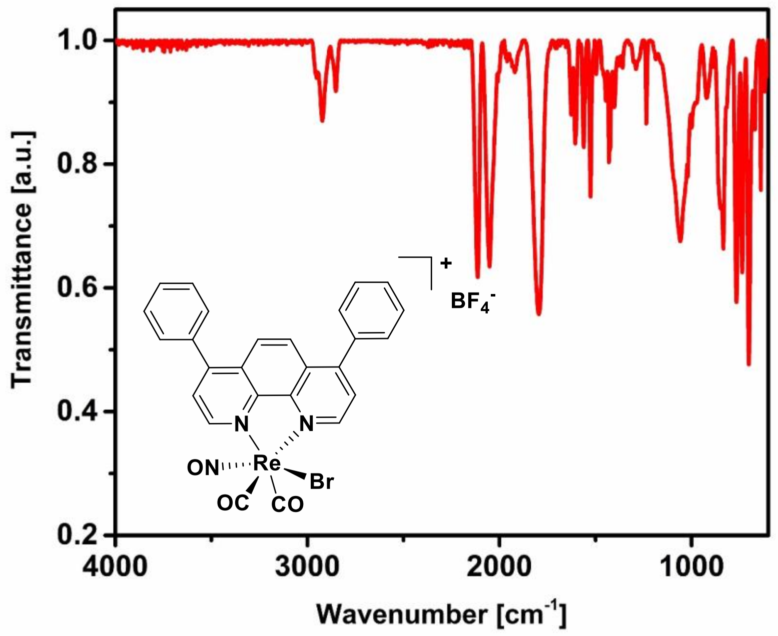

Figure S17. IR spectrum of [Re(CO)$_2$NO(φ-phen)Br](BF$_4$) (**5**).

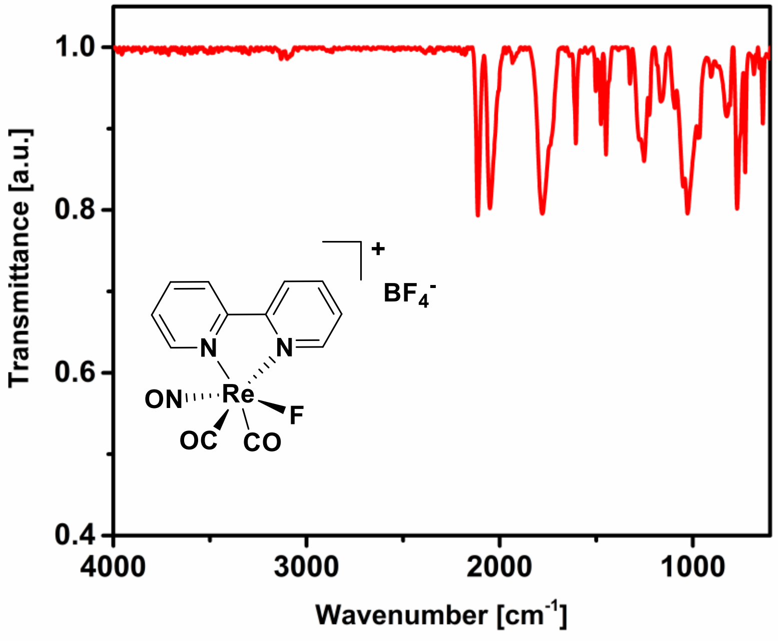

Figure S18. IR spectrum of [Re(CO)$_2$NO(bpy)F](BF$_4$) **(6).**

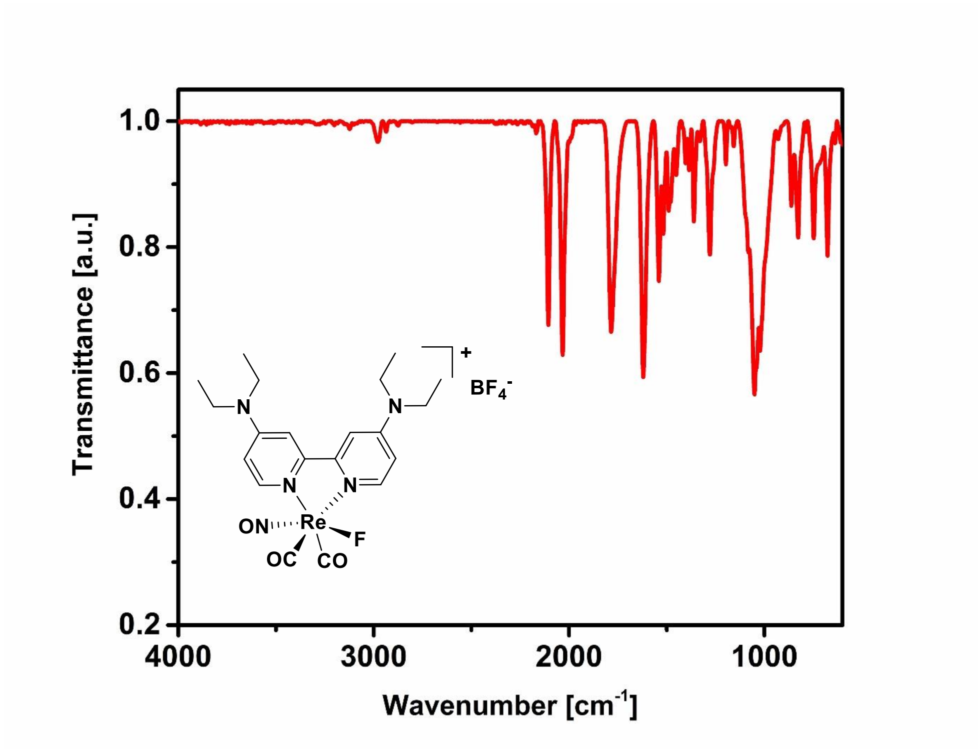

Figure S19. IR spectrum of [Re(CO)$_2$NO(Et$_2$N-bpy)F](BF$_4$) **(7)**.

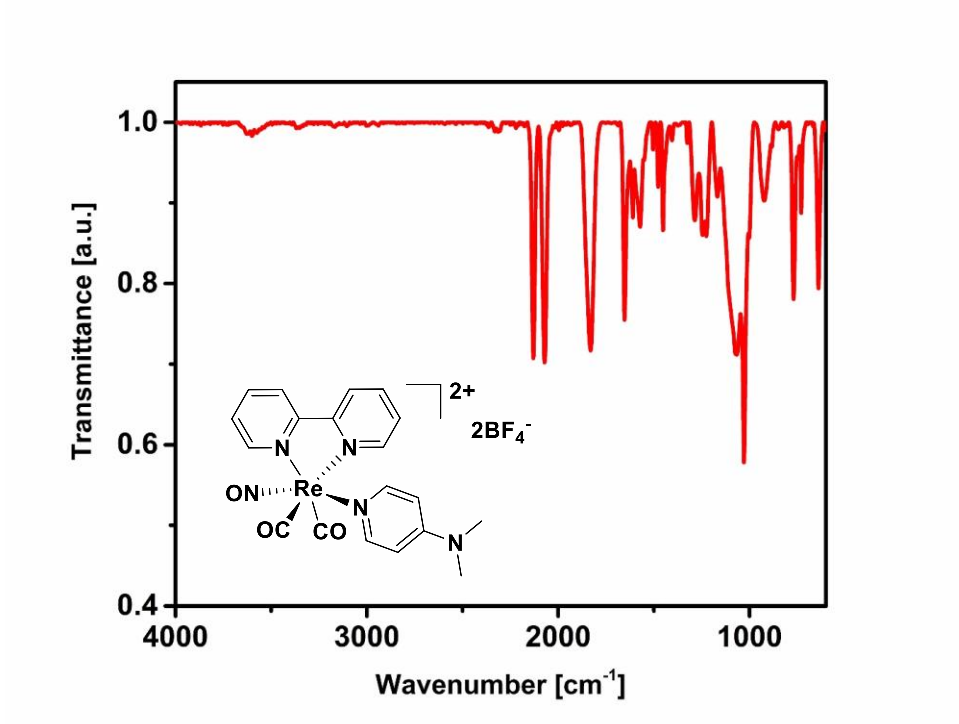

Figure S20. IR spectrum of [Re(CO)$_2$NO(bpy)(Me2N-py)](BF$_4$)$_2$ **(9)**.

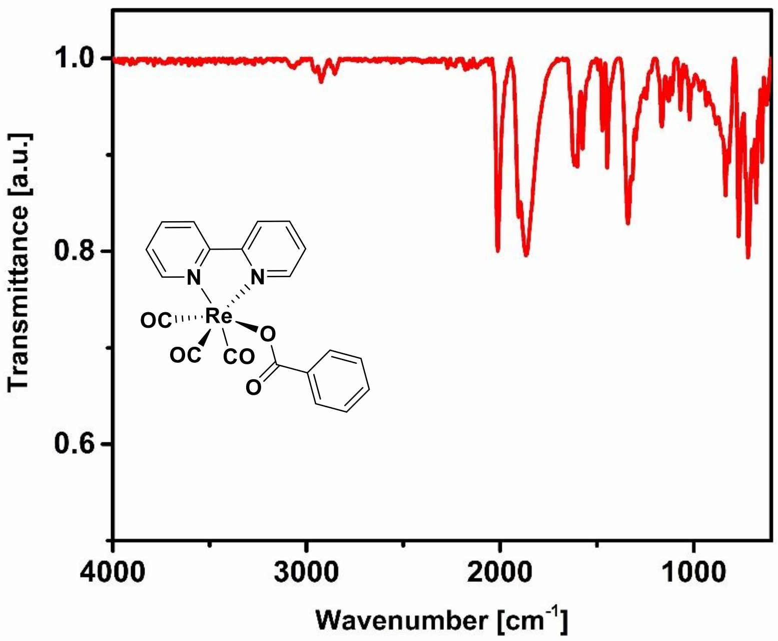

Figure S21. IR spectrum of [Re(CO)$_3$(bpy)(O$_2$CBz)] **(10)**.

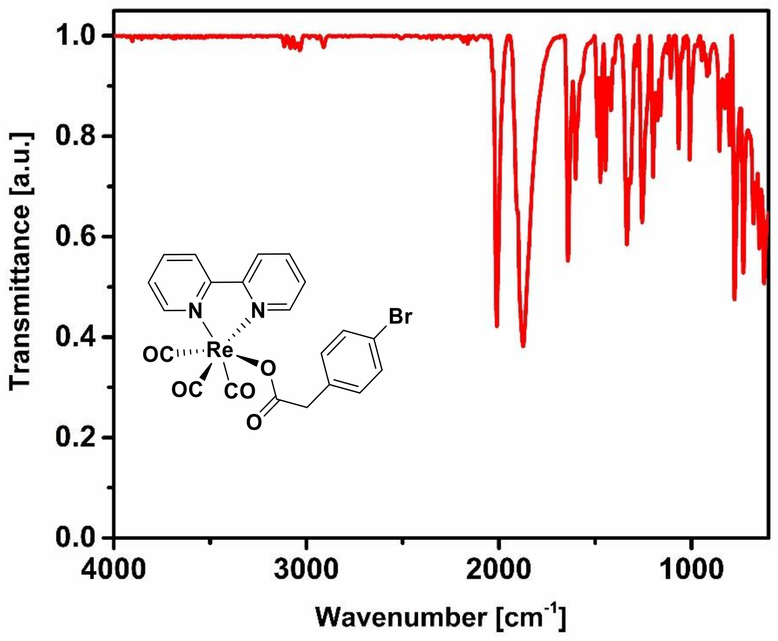

Figure S22. IR spectrum of [Re(CO)$_3$(bpy)(O$_2$CPh)] **(11)**.

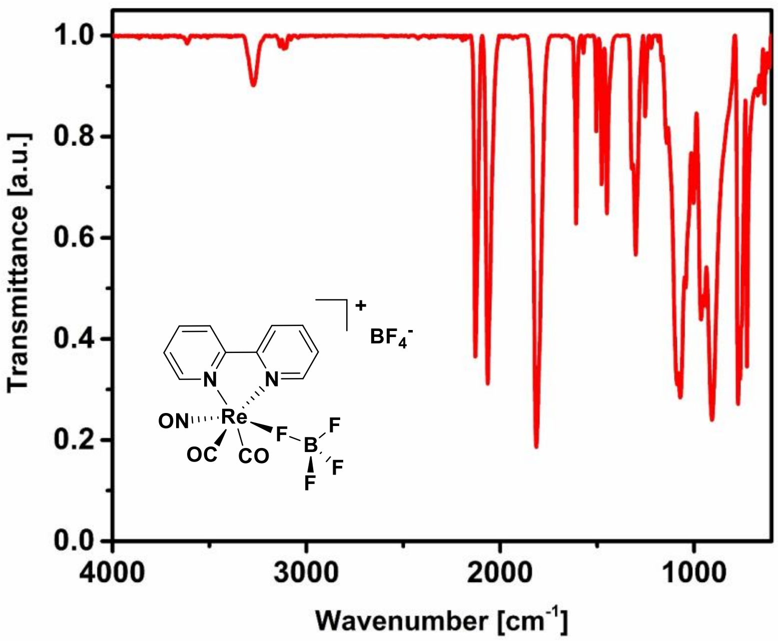

Figure S23. IR spectrum of $[Re(CO)_2NO(bpy)BF_4](BF_4)$ **(12)**.

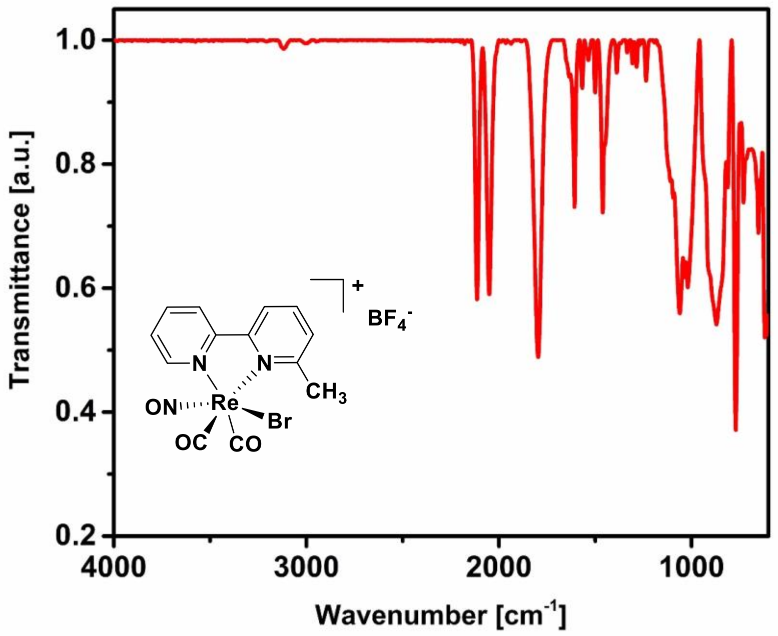

Figure S24. IR spectrum of $[Re(CO)_2NO(6\text{-}Me\text{-}bpy)Br](BF_4)$ (**14**).

UV-Vis spectra

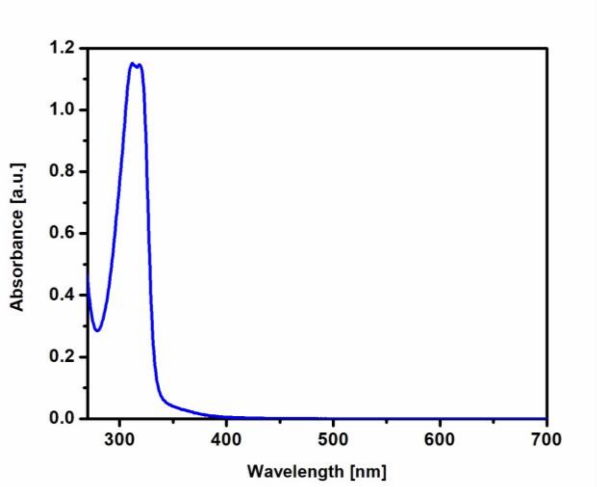

Figure S25. UV-Vis spectrum of $[Re(CO)_2NO(bpy)Br](BF_4)$ (**1**) in acetonitrile.

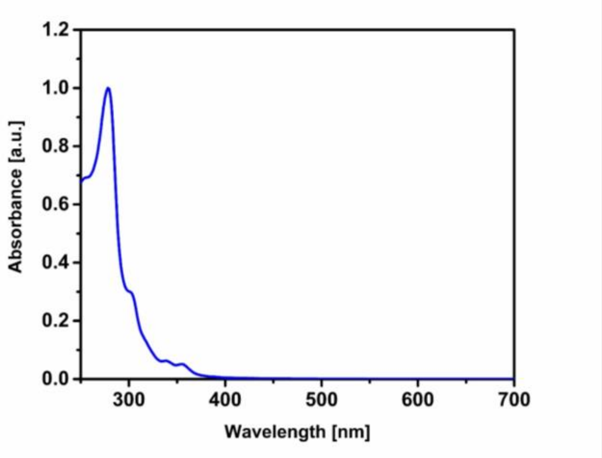

Figure S26. UV-Vis spectrum of $[Re(CO)_2NO(phen)Br](BF_4)$ (**2**) in DMF.

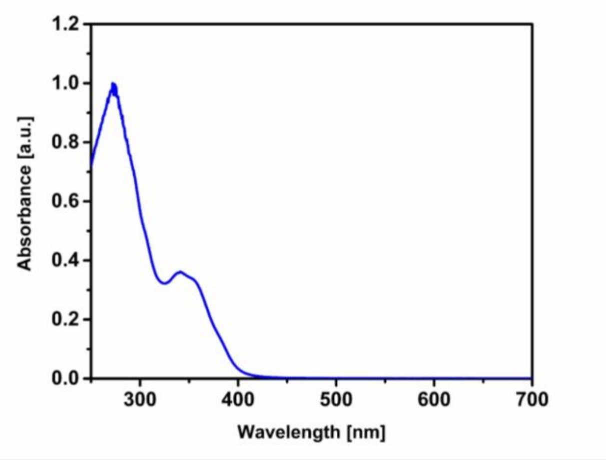

Figure S27. UV-Vis spectrum of $[Re(CO)_2NO(Et_2N\text{-}bpy)Br](BF_4)$ (**3**) in acetonitrile.

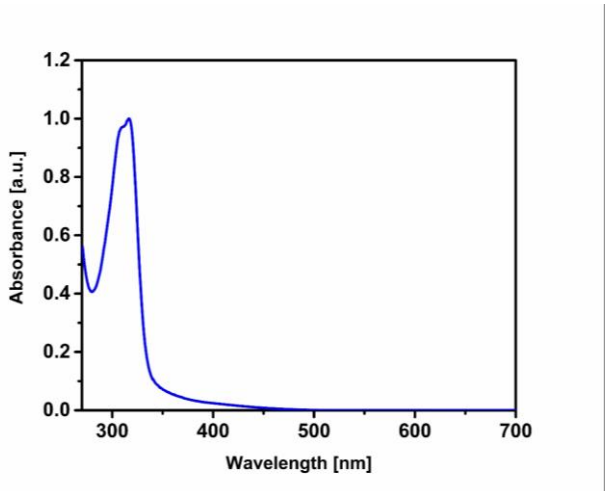

Figure S28. UV-Vis spectrum of $[Re(CO)_2NO(tBu\text{-}bpy)Br](BF_4)$ (**4**) in DMF.

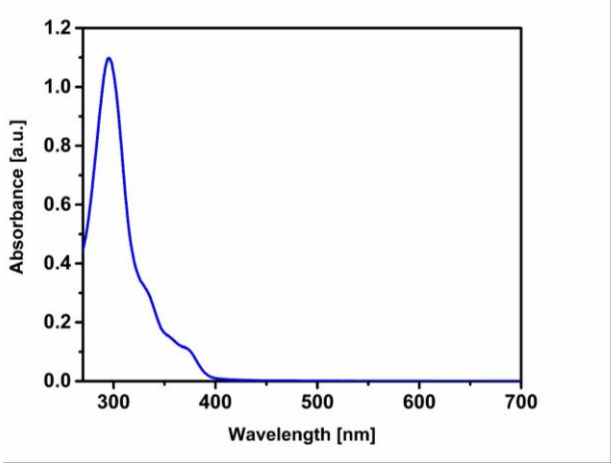

Figure S29. UV-Vis spectrum of $[Re(CO)_2NO(\varphi\text{-}phen)Br](BF_4)$ (**5**) in acetonitrile.

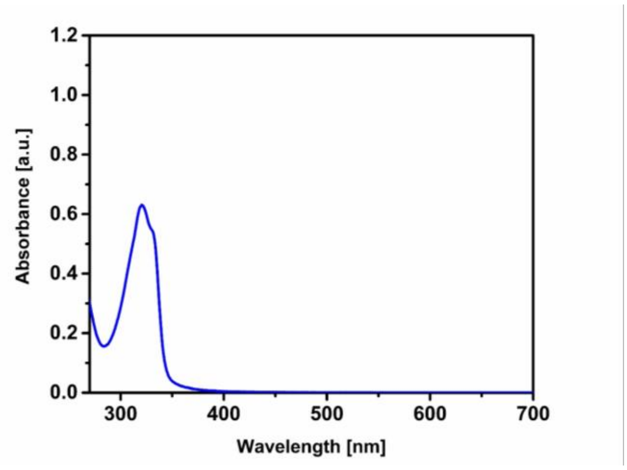

Figure S30. UV-Vis spectrum of $[Re(CO)_2NO(6\text{-}Me\text{-}bpy)Br](BF_4)$ (**14**) in acetonitrile.

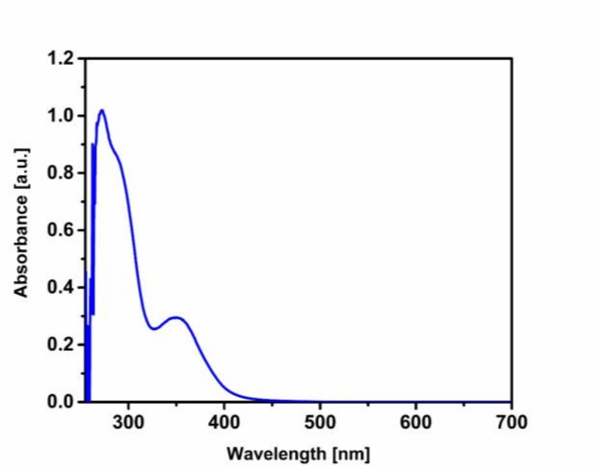

Figure S31. UV-Vis spectrum of [Re(CO)$_2$(NO)(Et$_2$N-bpy)F](BF$_4$) **(7)** in acetonitrile.

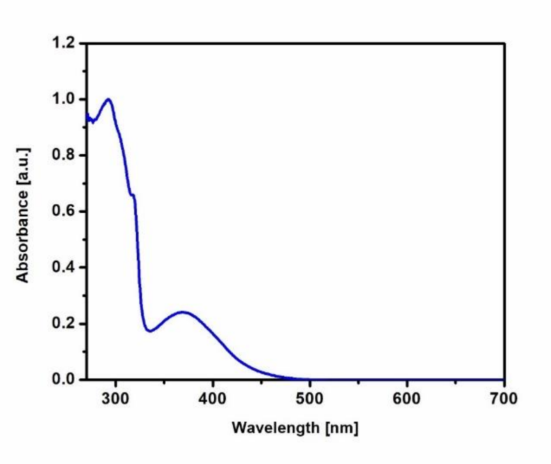

Figure S32. UV-Vis spectrum of [Re(CO)$_3$(bpy)(O$_2$CBz)] (**10**) in DMF.

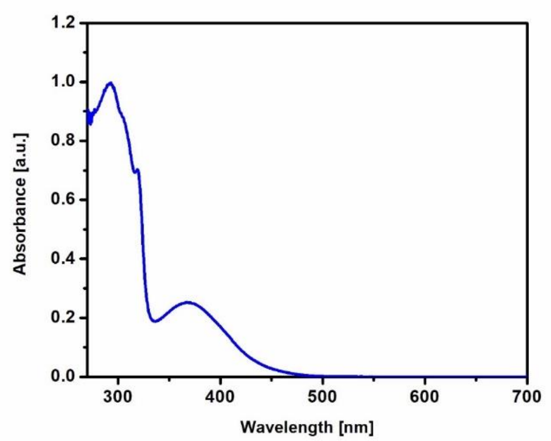

Figure S33. UV-Vis spectrum of [Re(CO)$_3$(bpy)(O$_2$CPh)] (**11**) in DMF.

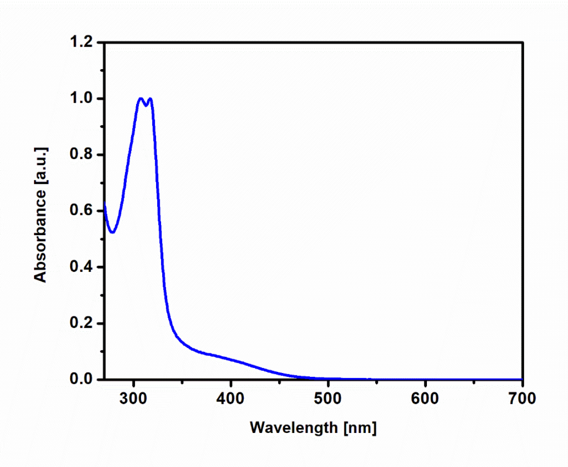

Figure S34. UV-Vis spectrum of [Re(CO)$_2$NO(bpy)BF$_4$](BF$_4$) **(12)** in DMF.

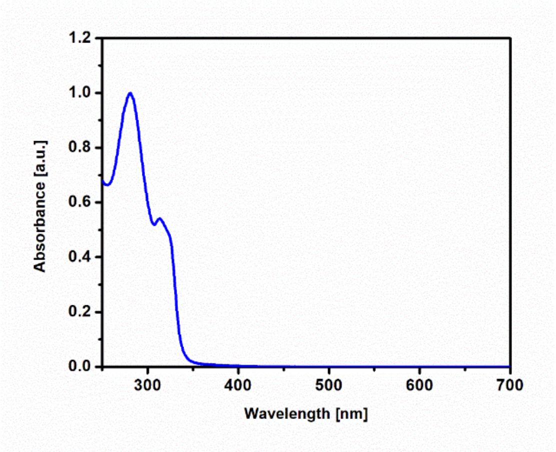

Figure S35. UV-Vis spectrum of [Re(CO)$_2$NO(bpy)(Me2N-py)](BF$_4$)$_2$ **(9)** in acetonitrile.

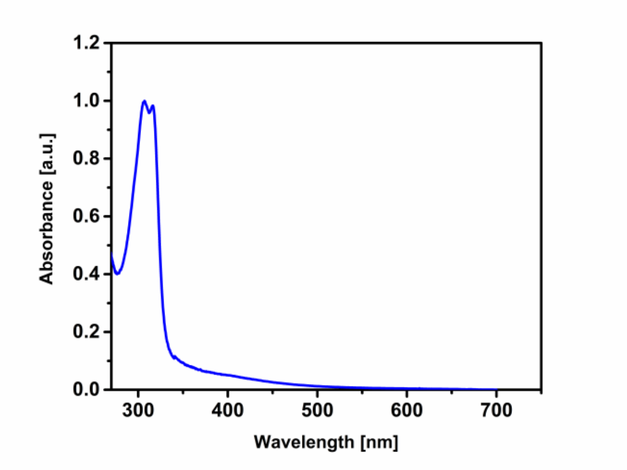

Figure S36. UV-Vis spectrum of [Re(CO)$_2$(NO)(bpy)F](BF$_4$) **(6)** in DMF.

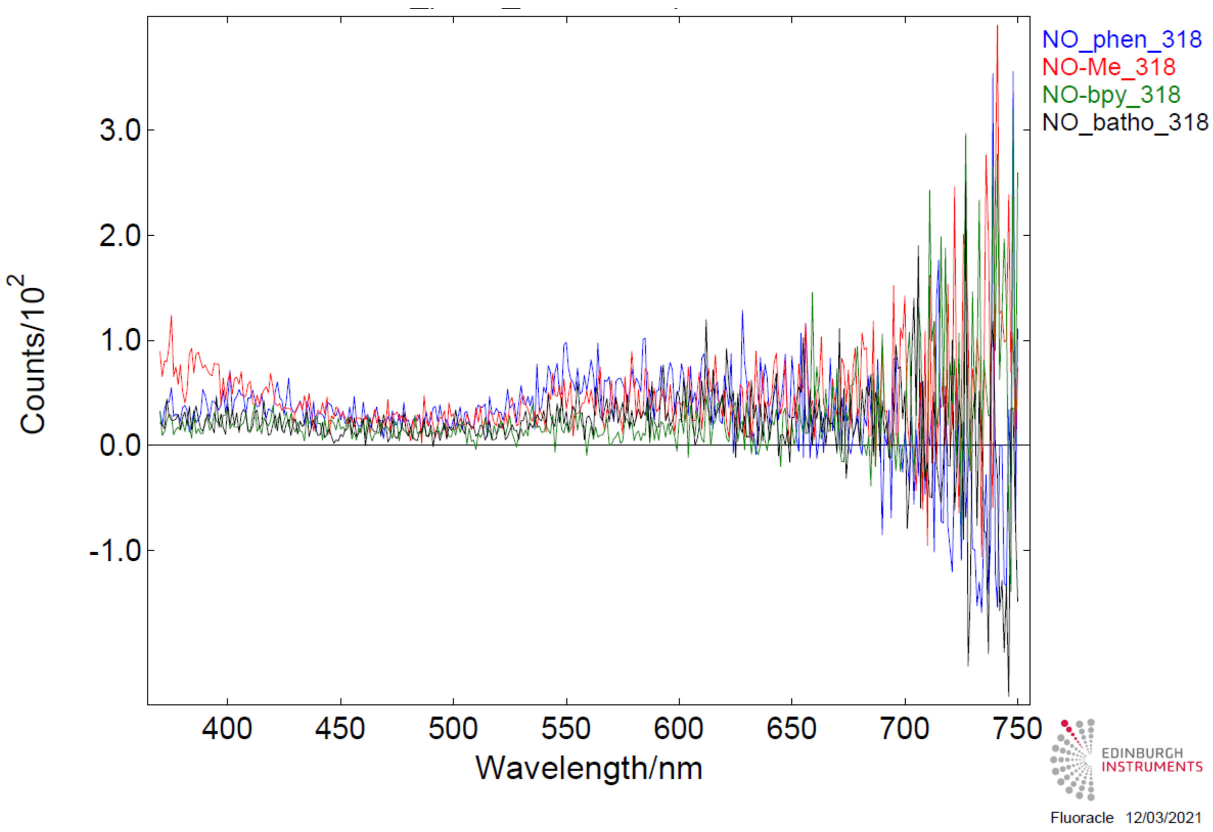

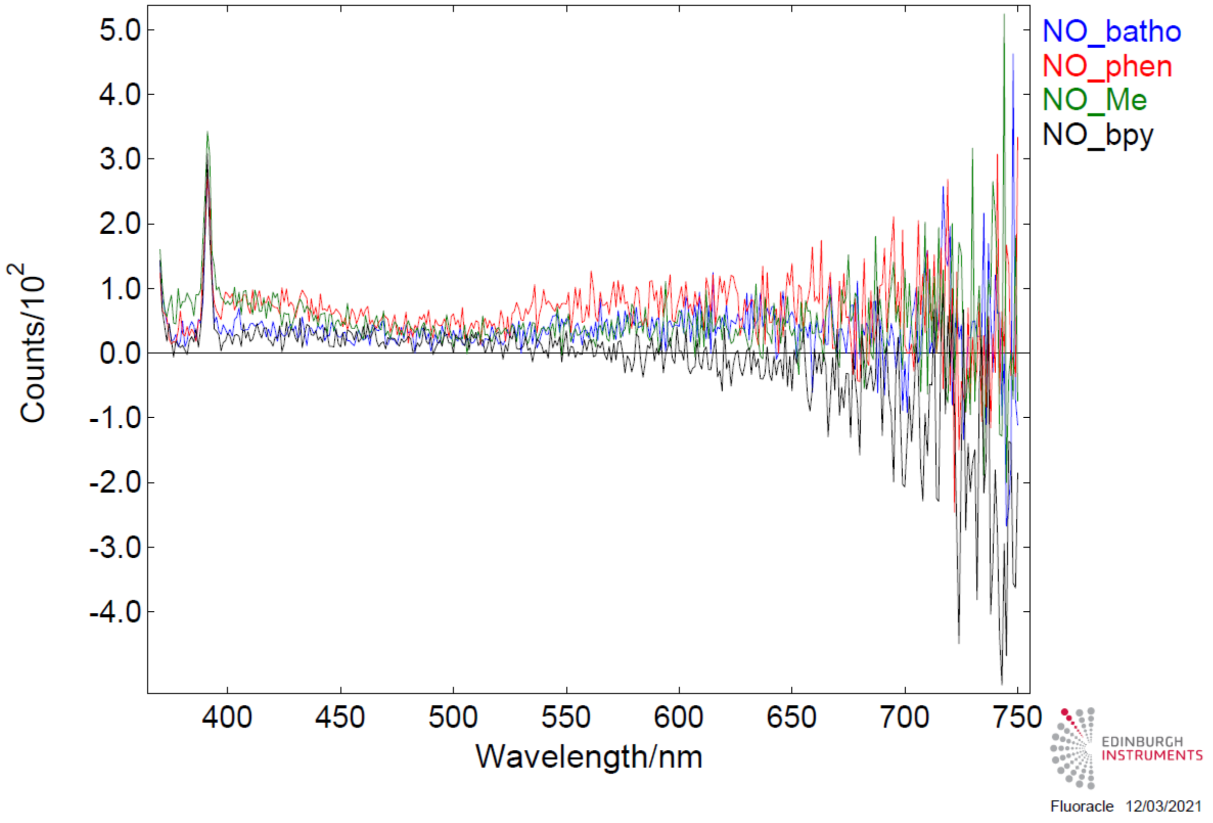

Figure S37. Emission spectra of selected nitrosyl Re complexes. Top: λ_{ex} = 318; bottom λ_{ex} = 350.

Table S1. Crystal data and structure refinement for **1**, **2**, **6-8**, **10-14,** and fully oxidized complex **14**.

Identification code	**1**	**2**	**6**	**7**	**8**	**10**	**11**	**12**	**13**	**14**	ox'd **14**
CSD number	2093631	2093632	2094070	2093633	2093634	2093635	2093636	2093637	2093638	2093639	2093640
Empirical formula	$C_{12}H_8BBrF_4N_3O_3Re$	$C_{14}H_8BBrF_4N_3O_3Re$	$C_{12}H_8BF_5N_3O_3Re$	$C_{20}H_{26}BF_5N_5O_3Re$	$C_{34}H_{26}Br_2N_6O_4Re_2$	$C_{21}H_{14}BrN_2O_5Re$	$C_{20}H_{13}N_2O_5Re$	$C_{12}H_{10}B_2F_8N_3O_4Re$	$C_{14}H_{11}B_3F_{12}N_4NaO_3Re$	$C_{13}H_{10}BBrF_4N_3O_3Re$	$C_{11}H_{11}N_2O_4Re$
Formula weight	595.13	619.15	534.22	676.47	1114.83	640.45	547.52	620.05	752.89	609.16	421.42
Temperature/K	200(2)	200(2)	250(2)	200(2)	200(2)	200(2)	200(2)	250(2)	200(2)	200(2)	200(2)
Crystal system	orthorhombic	monoclinic	monoclinic	monoclinic	orthorhombic	monoclinic	triclinic	monoclinic	triclinic	triclinic	orthorhombic
Space group	*P*nma	*P*2$_1$/c	*P*2$_1$/n	*P*2$_1$/n	*P*na2$_1$	*P*2$_1$/c	*P*-1	*P*2$_1$/n	*P*-1	*P*-1	*P*nma
a/Å	17.0900(4)	10.6603(3)	10.0421(2)	9.26150(10)	16.1750(2)	20.1830(6)	8.4295(4)	7.4804(2)	8.6774(3)	7.0101(4)	9.5298(4)
b/Å	12.1181(2)	12.4496(3)	7.8180(2)	23.2899(3)	8.35450(10)	10.7028(2)	9.4918(4)	24.3398(7)	10.8057(4)	10.9539(4)	6.6170(3)
c/Å	8.01100(10)	13.2172(4)	20.2281(4)	12.5774(2)	25.8524(3)	9.4252(3)	12.7023(6)	11.1812(2)	13.1764(4)	11.3985(7)	18.6332(11)
α/°	90	90	90	90	90	90	106.704(3)	90	75.144(3)	84.168(3)	90
β/°	90	105.443(2)	98.0260(10)	110.6400(10)	90	97.923(2)	92.147(4)	109.070(2)	73.474(3)	77.822(5)	90
γ/°	90	90	90	90	90	90	112.452(3)	90	89.797(3)	84.140(4)	90
Volume/Å^3	1659.06(5)	1690.81(8)	1572.54(6)	2538.80(6)	3493.54(7)	2016.55(10)	887.12(7)	1924.05(9)	1141.65(7)	848.21(8)	1174.99(10)
Z	4	4	4	4	4	4	2	4	2	2	4
ρ_{calc}g/cm^3	2.383	2.432	2.256	1.770	2.120	2.110	2.050	2.141	2.190	2.385	2.382
μ/mm^{-1}	17.733	17.442	15.833	9.977	16.411	14.433	13.724	13.323	11.792	17.364	20.343
F(000)	1104.0	1152.0	1000.0	1320.0	2096.0	1216.0	524.0	1168.0	712.0	568.0	792.0
2Θ range for data collection/°	10.352 to 135.344	8.606 to 136.05	9.362 to 135.072	7.592 to 136.81	10.94 to 135.768	8.848 to 135.636	10.67 to 129.818	13.102 to 135.856	7.26 to 135.066	13.778 to 129.57	10.426 to 134.826
*R*eflections collected	20797	17444	41477	38156	34006	3542	21038	18854	36017	15094	4916
Independent reflections	1573 [R_{int} = 0.0336, R_{sigma} = 0.0124]	2947 [R_{int} = 0.0386, R_{sigma} = 0.0170]	2801 [R_{int} = 0.0280, R_{sigma} = 0.0082]	4497 [R_{int} = 0.0343, R_{sigma} = 0.0159]	4908 [R_{int} = 0.0294, R_{sigma} = 0.0127]	3542 [R_{int} = ?, R_{sigma} = 0.0151]	2904 [R_{int} = 0.0509, R_{sigma} = 0.0238]	3229 [R_{int} = 0.0529, R_{sigma} = 0.0237]	3817 [R_{int} = 0.0677, R_{sigma} = 0.0229]	2750 [R_{int} = 0.0278, R_{sigma} = 0.0129]	1082 [R_{int} = 0.0236, R_{sigma} = 0.0121]
Data/restraints/parameters	1573/0/129	2947/0/245	2801/0/226	4497/0/321	4908/1/434	3542/110/273	2904/0/253	3229/36/299	3817/0/343	2750/0/237	1082/1/108
Goodness-of-fit on F^2	1.192	1.061	1.290	1.109	1.101	1.167	1.123	1.082	1.102	1.149	1.085
Final *R* indexes [I>=2σ (I)]	R_1 = 0.0248, wR_2 = 0.0700	R_1 = 0.0260, wR_2 = 0.0738	R_1 = 0.0289, wR_2 = 0.0647	R_1 = 0.0309, wR_2 = 0.0873	R_1 = 0.0250, wR_2 = 0.0654	R_1 = 0.0686, wR_2 = 0.1786	R_1 = 0.0312, wR_2 = 0.0793	R_1 = 0.0439, wR_2 = 0.1020	R_1 = 0.0406, wR_2 = 0.1022	R_1 = 0.0234, wR_2 = 0.0626	R_1 = 0.0346, wR_2 = 0.1014
Final *R* indexes [all data]	R_1 = 0.0249, wR_2 = 0.0700	R_1 = 0.0267, wR_2 = 0.0745	R_1 = 0.0290, wR_2 = 0.0647	R_1 = 0.0334, wR_2 = 0.0888	R_1 = 0.0252, wR_2 = 0.0656	R_1 = 0.0703, wR_2 = 0.1795	R_1 = 0.0312, wR_2 = 0.0793	R_1 = 0.0473, wR_2 = 0.1041	R_1 = 0.0406, wR_2 = 0.1022	R_1 = 0.0234, wR_2 = 0.0626	R_1 = 0.0352, wR_2 = 0.1019
Largest diff. peak/hole / e Å^{-3}	0.95/-0.71	1.46/-1.02	1.25/-1.54	0.72/-1.00	0.81/-0.76	2.58/-2.83	0.72/-1.96	0.95/-0.93	2.04/-1.64	1.31/-1.55	1.55/-0.83
Flack parameter					0.429(14)						

Suitable crystal were selected and mounted on loop with oil on a Stoe StadiVari diffractometer. The crystal were kept at 200(2) K during data collection, excepted for **6** and **12** (250(2) K). Using Olex2 [1], the structure was solved with the SHELXT [2] structure solution program using Intrinsic Phasing and refined with the SHELXL [3] refinement package using Least Squares minimization.

1. Dolomanov, O.V., Bourhis, L.J., Gildea, R.J, Howard, J.A.K. & Puschmann, H. (2009), J. Appl. Cryst. 42, 339-341.
2. Sheldrick, G.M. (2015). Acta Cryst. A71, 3-8.
3. Sheldrick, G.M. (2015). Acta Cryst. C71, 3-8.
